# Automated synthesis of sialylated oligosaccharides

**DOI:** 10.3762/bjoc.8.183

**Published:** 2012-09-21

**Authors:** Davide Esposito, Mattan Hurevich, Bastien Castagner, Cheng-Chung Wang, Peter H Seeberger

**Affiliations:** 1 Max-Planck-Institute of Colloids and Interfaces, Department of Biomolecular Systems, Am Mühlenberg 1, 14476 Potsdam, Germany; 2 Freie Universität Berlin, Institute of Chemistry and Biochemistry, Arnimallee 22, 14195 Berlin, Germany; 3 Institute of Pharmaceutical Sciences, Swiss Federal Institute of Technology (ETH) Zurich, 8093 Zurich, Switzerland; 4 Institute of Chemistry, Academia Sinica, Taipei, 11529, Taiwan

**Keywords:** automated synthesis, disaccharide building blocks, solid-phase synthesis, sialic acid, sialosides

## Abstract

Sialic acid-containing glycans play a major role in cell-surface interactions with external partners such as cells and viruses. Straightforward access to sialosides is required in order to study their biological functions on a molecular level. Here, automated oligosaccharide synthesis was used to facilitate the preparation of this class of biomolecules. Our strategy relies on novel sialyl α-(2→3) and α-(2→6) galactosyl imidates, which, used in combination with the automated platform, provided rapid access to a small library of conjugation-ready sialosides of biological relevance.

## Introduction

Sialic acid (Sia) belongs to a family of nonulosonic acids, i.e., monosaccharides equipped with a carboxylic moiety and a nine-carbon backbone, which play a unique role in glycobiology. Sia-containing glycans mediate pathogen invasion [1] and are involved in signalling cascades, which have been extensively studied [2]. The distinctive structure of Sia confers special properties to membrane oligosaccharides [3] resulting in sialosides having exceptional biological significance. Rapid access to synthetic sialylated glycans would contribute greatly to the biological studies on this important class of molecules. The automated synthesis of oligosaccharides has been significantly improved since the first report in 2001 [4]. Currently, the platform enables the rapid assembly of complex oligosaccharides and accommodates the most commonly employed glycosylation reactions [5–7]. However, accessing sialosides by automation has been hampered by several factors. Chemical silalylation represents a significant challenge, and is usually plagued by low yields and anomeric mixtures [8]. To avoid synthetic complications, Sia has often been introduced by enzymatic methods [9]. In order to allow for access to synthetic sialosides, an intense effort has been devoted to identifying sialic acid building blocks with superior sialylation properties [10]. In turn, only limited attention has been given to the design of more efficient nucleophiles for sialylations. In naturally occurring *N*- and *O*-glycans the terminal sialic acid residue is most often connected to the C3 or C6 hydroxy group of galactose. Therefore, differently protected galactose precursors have been exploited for sialylation reactions. The obtained disaccharides have been used to prepare synthetic sialosides [11–13]. A disaccharide building block approach is an attractive possibility for solid-phase synthesis, since it avoids performing a low yielding and unselective sialylation on a solid support.

Here, we describe a method for the rapid preparation of different sialosides relying on a new automated solid-phase synthesis platform [5]. Central to the success of this approach is the use of galactals as nucleophiles for chemical sialylation, which allows for efficient access to the novel sialyl α-(2-3) and α-(2-6) galactosyl imidate disaccharide building blocks. The combination of the automated platform and sialylated building blocks proved successful for the synthesis of representative Sia-containing oligosaccharides ready for biological evaluation.

## Results and Discussion

### Building-blocks preparation

**Figure 1:**
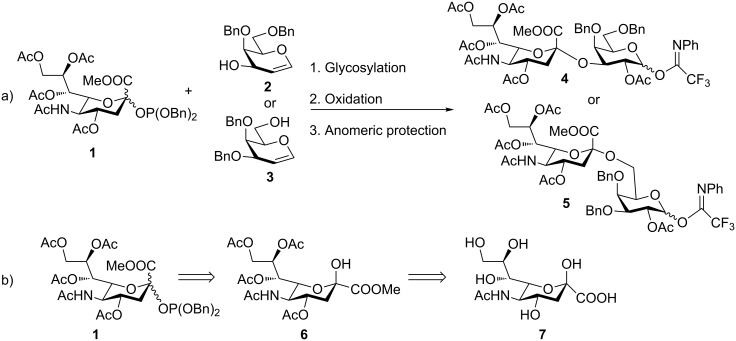
(a) Synthesis sequence for the preparation of building blocks **4** and **5**; (b) Retrosynthetic analysis for the preparation of **1**.

Many sialylation strategies utilize building blocks that require multistep syntheses [10]. In contrast, solid-phase automated synthesis requires readily accessible building blocks that can be used in excess to drive reactions to completion. As our initial goal, we developed a method to provide sialic acid containing disaccharide glycosylating agents with minimal synthetic effort. Simple *N*-acetyl building blocks such as **1** (Figure 1) were used due to their facile syntheses, in contrast to other commonly employed *N*-5 modified building blocks [14–17]. Most of the *N-*acetyl sialic acid glycosylating agents reported in the literature can be accessed from the common intermediate **6** [18], which is prepared in two steps from commercially available Sia (Figure 1b).

Compound **6** was converted in a single step into various sialylating agents, such as the *N-*phenyl trifluoroacetimidoyl glycoside **10**, and glycosyl phosphites **8** and **1** as previously described (Table 1) [19–21]*.* Galactal **2** was identified recently as an efficient acceptor for sialylation [11–12]. Its efficiency can be attributed to a combination of reduced steric hindrance and good nucleophilicity of the C3 hydroxy group. Thus, galactal **2** was glycosylated with different *N*-acetyl sialic acid building blocks (Table 1).

**Table 1:** Synthesis of building blocks **9** and **11**.

entry	glycosylating agent	nucleophile	conditions^a^	product	yield	α/β ratio

**1**	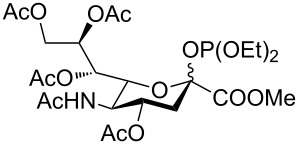 **8** (1.0 equiv)	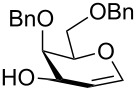 **2** (1.5 equiv)	a	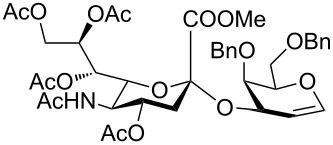 **9**	–	–
**2**	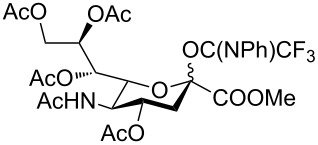 **10** (1.0 equiv)	**2** (1.5 equiv)	a	**9**	30%	9/1
**3**	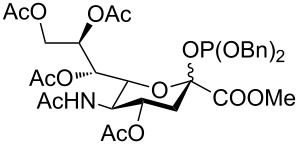 **1** (1.0 equiv)	**2** (1.5 equiv)	a	**9**	27%	9/1
**4**	**1** (1.0 equiv)	**2** (1.7 equiv)	b	**9**	80%	4/1
**5**	**1** (1.0 equiv)	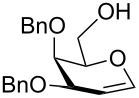 **3** (2.0 equiv)	b	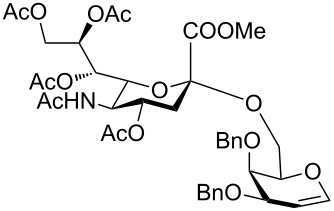 **11**	75%	2.5/1

^a^Reagents and Conditions: (a) TMSOTf (0.2 equiv), EtCN, AW-4Å MS, −78 °C; (b) TMSOTf (0.2 equiv), CH_3_CN, AW-4Å MS, −42 °C.

We started our screening by comparing the glycosylation of building blocks **1**, **8** and **10** with galactal **2** under similar conditions (Table 1, entries 1–3). Sialylation with building block **8** failed to yield any disaccharide **9** (Table 1, entry 1), while glycosylating agents **10** and **1** gave moderate yields and good selectivity (Table 1, entries 2 and 3). The glycosylation of galactal **2** with phosphite **1**, which we described in the supporting information of [5], was further optimized. In particular, elevating the reaction temperature proved beneficial and disaccharide **9** was isolated in higher overall yield, albeit with a slight decrease in selectivity. The best results were obtained by using 1.7 equivalents of **2** at −42 °C, using acetonitrile as the solvent instead of the more expensive propionitrile (Table 1, entry 4). The conditions established for the synthesis of compound **8** were applied to synthesize sialyl α-(2→6) galactal **11** (Table 1, entry 5) in good yield upon glycosylation of galactal **3** [22]. In all cases, the desired anomer was readily purified by column chromatography.

**Scheme 1:**
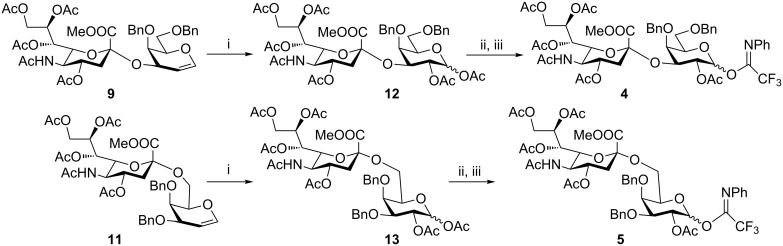
Reagents and conditions: (i) PhI(OAc)_2,_ BF_3_·Et_2_O, CH_2_Cl_2,_ −40 °C; then Ac_2_O, pyridine; (ii) N_2_H_4_·AcOH, DMF; (iii) CF_3_C(NPh)Cl, Cs_2_CO_3_, CH_2_Cl_2_, DCM, 66% over three steps for **4** (for a detailed description of the synthesis of compound **4** see the supporting information of [5]); 62% over three steps for **5**.

In order to convert the disaccharide products into the corresponding glycosyl imidates, the double bond in compounds **9** and **11** was oxidized by treatment with PhI(OAc)_2_ and catalytic amounts of BF_3_·Et_2_O [23] and gave disaccharides **12** and **13**, respectively after acetylation (Scheme 1). Removal of the anomeric acetate mediated by hydrazine acetate provided the hemiacetals, which was followed by introduction of the anomeric *N*-phenyl trifluoroacetimidate to furnish disaccharide building blocks **4** and **5**. It should be noted that building block **4** can be prepared with higher overall yield than the recently disclosed *N-*Troc protected disaccharide building block [11] obtained with a similar method.

### Solution-phase studies

**Scheme 2:**
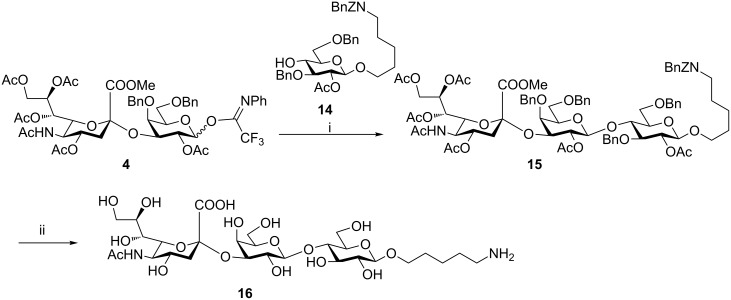
Reagents and conditions. (i) TMSOTf, DCM, −10 °C, 80%; (ii) NaOMe, MeOH; then KOH, MeOH, 60 °C; then Pd/C, H_2_, AcOH, MeOH, THF, H_2_O, rt, 76% over three steps.

In order to evaluate the utility of building block **4** for the solid-phase synthesis of sialosides we undertook a model solution-phase synthesis of the glycan portion of GM3 ganglioside **16** (Scheme 2). GM3 serves as an important receptor for viral infection [24–25] and contains the common sialyl α-(2→3) galactose motif. The key step en route to compound **16** was the glycosylation of compound **14** with building block **4** (Scheme 2), which proceeded efficiently in the presence of trimethylsilyl triflate (TMSOTf) as promoter at −10 °C to afford trisaccharide **15** with a yield of 80%. It is worth mentioning that glycosylation of an analogue of glucose **14** equipped with a benzoyl group at the C3 hydroxy position resulted in a lower glycosylation yield (36%), suggesting that an ester can lower the nucleophilicity of the vicinal C4-hydroxy. The synthesis was completed by deacetylation of compound **15** under Zemplén's conditions, followed by saponification and hydrogenolysis affording good yields of the trisaccharide **16**, equipped with an amino spacer for conjugation. The synthesis of GM3 trisaccharide **16** proved that compound **4** is efficient for installing the capping sialyl α-(2→3) galactose unit into synthetic oligosaccharides. Furthermore, conditions applied to the preparation of **16** can be easily adapted for solid-phase synthesis making **4** a valuable candidate for automation.

### Automated synthesis of sialosides

#### Automated synthesis of linear α-(2→3) sialosides

**Scheme 3:**
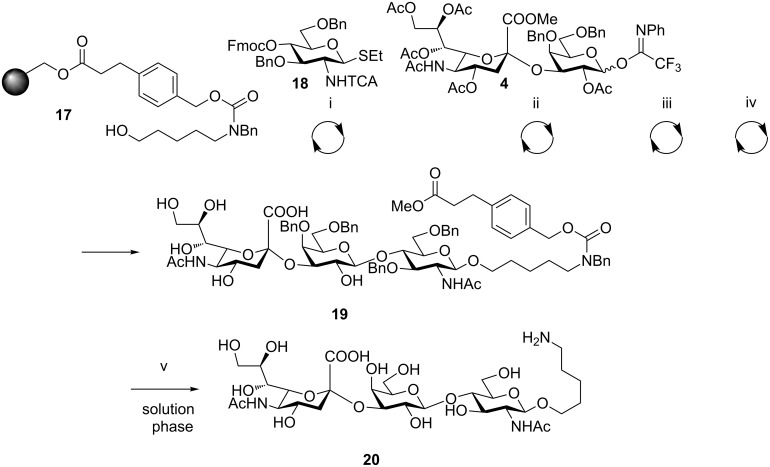
Automated synthesis of **20**. Reagents and conditions: (i) (a) NIS, TfOH, dioxane, DCM, −40 to −20 °C, 40 min; (b) piperidine, DMF. (ii) (a) TMSOTf, DCM, 0 °C, 2 h; (b) piperidine, DMF. (iii) AIBN (cat.), Bu_3_SnH (10 equiv), xylene, 90 °C. (iv) NaOMe, MeOH, DCM, 1.5 h, 33%. (v) Pd/C, H_2_, MeOH/H_2_O, cat. AcOH, 78% (for experimental details see the supporting information of [5]).

**Scheme 4:**
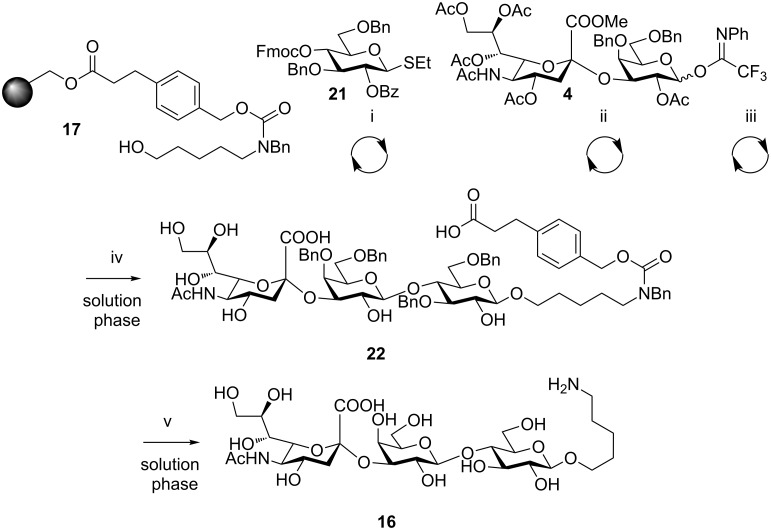
Automated synthesis of **16**. Reagents and conditions: (i) (a) NIS, TfOH, dioxane, DCM, −40 to −20 °C, 40 min; (b) piperidine, DMF. (ii) (a) TMSOTf, DCM, 0 °C, 2 h; (b) piperidine, DMF. (iii) NaOMe, MeOH, DCM, 1.5 h. (iv) KOH, MeOH, H_2_O, THF, 60 °C, 40%. (v) Pd/C, H_2_, MeOH/H_2_O, cat. AcOH, 91% (for experimental details see the supporting information of [5]).

The new integrated platform for automated synthesis of oligosaccharides [5] offers the possibility to construct a diverse set of glycans rapidly and efficiently. The automated synthesizer proved capable of performing iterative glycosylation–deprotection cycles under conditions commonly employed for solution-phase oligosaccharide synthesis. The synthetic strategy relies on the solid support-bound linker **17** (Scheme 3), which contains a latent amino spacer useful for conjugation. In addition, manual operations are minimized by performing the trichloroacetyl (TCA) reduction, ester removal and cleavage from the solid support by automation. In many cases, only hydrogenolytic cleavage of the remaining benzyl ethers and carbamates has to be performed manually at the end of an automated sequence. These features make the automated platform very attractive for sialoside synthesis. Based on the encouraging results obtained for the solution-phase synthesis of **16**, building blocks **4** and **5** were used for the automated solid-phase synthesis of various sialosides. Some of the results presented herein have been communicated in preliminary form [5]. Sialyl lactosamine **20** (Scheme 3) [25], which serves as a site of attachment for viruses during infections, and sialyl lactose (GM3) **16** (Scheme 4) were chosen to confirm the viability of building block **4** for the automated solid-phase synthesis of linear sialosides.

For all sialosides, a similar synthetic route was followed, consisting of automated glycosylation and deprotection cycles, followed by TCA reduction (when glucosamines are present) and final ester removal/cleavage to afford the semi-protected oligosaccharide. The automated synthesis of **20** (Scheme 3) started with the glycosylation of resin-bound linker **17** with glucosamine building block **18** (2 × 5 equiv) [5] in the presence of *N*-iodosuccinimide and triflic acid. Fluorenylmethoxycarbonyl (Fmoc) removal was followed by glycosylation with building block **4** (2 × 5 equiv) for 1 h at −10 °C with TMSOTf used for activation. Radical reduction using tributyltinhydride and azobisisobutyronitrile (AIBN) was performed to convert the trichloroacetamide into an *N*-acetyl moiety, followed by methoxide-mediated cleavage to provide compound **19**. Analysis of the crude mixture by LC–MS showed incomplete glycosylation of the resin bound glucosamine by building block **4.** The reaction was optimized to identify the best glycosylation conditions for building block **4** under the solid-phase paradigm. Performing the glycosylation at a higher temperature (0 °C) and longer time (2 h) proved sufficient to drive the reaction to higher conversion and trisaccharide **19** was isolated in 33% overall yield with respect to resin loading. Hydrogenolysis under standard conditions afforded the fully deprotected trisaccharide **20** in 78% yield. These conditions were applied to the synthesis of GM3 trisaccharide **16** previously prepared in solution phase (see above*)*. Glucose thioglycoside building block **21** and disaccharide building block **4** served for the assembly of **16** (Scheme 4). Final saponification afforded the partially protected glycan **22** in 40% overall yield before hydrogenolysis gave the final trisaccharide **16** in 91% yield. The efficiency of the solid-phase and the solution-phase syntheses was compared. The solution-phase synthesis of trisaccharide **16** was completed with an overall yield of 42% (taking into account the preparation of compound **14**, 69%) in about one week. The same number of steps was executed in the solid-phase synthesis with little operator interference, to yield the desired compound in a comparable overall yield (36%) in shorter time. An average time of 3 h per glycosylation cycle (coupling and deprotection) or cleavage from the support, allows the assembly of a trisaccharide in roughly 10 h. In general, although an excess of building block is used in the automated solid-phase synthesis, the method provides the final assembled oligosaccharide with much greater efficiency than in the solution-phase synthesis, and avoids the loss of material encountered when performing purifications in between steps.

#### Automated synthesis of sialyl Lewis^X^

Branching is often observed in naturally occurring sialosides. Assembly of branched oligosaccharides is particularly challenging due to the steric hindrance of the branching sites, which can affect glycosylation yields. Working in a solid-phase environment could, in principle, additionally reduce the accessibility of a sterically hindered nucleophile. Thus, the possibility of accessing branched structures was explored on the solid support [5].

**Scheme 5:**
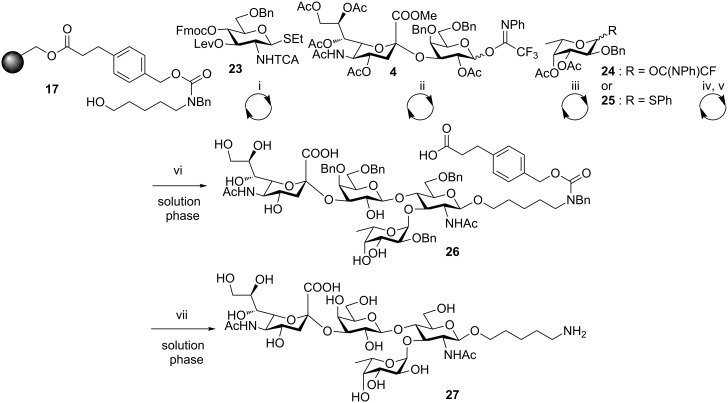
Automated synthesis of **27**. Reagents and conditions: (i) (a) NIS, TfOH, dioxane, DCM, −40 to −20 °C, 40 min; (b) piperidine, DMF. (ii) (a) TMSOTf, DCM, 0 °C, 2 h; (b) NH_2_NH_2_·H_2_O, AcOH, pyridine, DCM. (iii with **24**) TMSOTf, Et_2_O, −10 °C, 1 h. (iii with **25**) NIS, TfOH, dioxane, DCM, −40 °C to −20 °C, 40 min. (iv) AIBN (cat.), Bu_3_SnH (10 equiv), xylene, 90 °C. (v) NaOMe, MeOH, DCM, 1.5 h. (vi) KOH, MeOH, H_2_O, THF, 60 °C, 51%. (vii) Pd/C, H_2_, MeOH/H_2_O, cat. AcOH, 30% (for experimental details see the supporting information of [5]).

Sialyl Lewis^X^ tetrasaccharide **27** (Scheme 5), has been implicated in inflammation and cancer metastasis [26], and was chosen as a model glycan for the construction of branched compounds. Glucosamine building block **23,** containing C3-levulinoyl (Lev) and C4-Fmoc protecting groups [27], was first reacted with the linker. The glycosylation was followed by Fmoc removal from the C4 hydroxy group and a second glycosylation was performed with building block **4** under the conditions optimized in the context of the synthesis of trisaccharide **20**. Removal of the levulinoyl ester from C3 by treatment with hydrazine hydrate and acetic acid exposed the second hydroxy nucleophile on the central glucosamine. Our first attempt to glycosylate using fucose thioglycoside building block **25** afforded the product in low yield and as a mixture of anomers as confirmed by LC–MS analysis. The use of *N*-phenyl trifluoroacetimidate building block **24** proved more efficient. Nevertheless, when fucosylation with building block **24** was performed in dichloromethane, a mixture of anomers of compounds **26** was detected by NMR analysis. Only running the reaction in ether, which is a strong α-directing solvent [28], ensured stereoselective introduction of the fucose residue. Under these optimized conditions, the branched tetrasaccharide **26** was isolated in 51% overall yield after TCA reduction, cleavage, ester saponification and HPLC purification. Finally, solution-phase hydrogenolysis gave tetrasaccharide **27**.

#### Automated synthesis of linear α–(2→6) sialosides

**Scheme 6:**
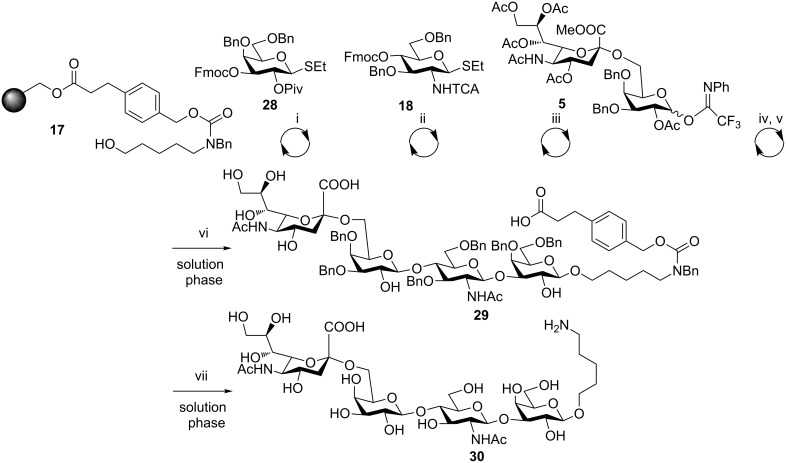
Automated synthesis of **30**. Reagents and conditions: (i) (a) NIS, TfOH, dioxane, DCM, −40 to −20 °C, 40 min; (b) piperidine, DMF. (ii) NIS, TfOH, dioxane, DCM, −40 to −20 °C, 40 min. (iii) TMSOTf, Et_2_O, 0 °C, 2 h. (iv) AIBN (cat.), Bu_3_SnH (10 equiv), xylene, 90 °C. (v) NaOMe, MeOH, DCM, 1.5 h. (vi) KOH, MeOH, H_2_O, THF, 60 °C, 16%. (vii) Pd/C, MeOH/H_2_O/EtOAc, cat. AcOH, 51%.

α-(2→6) Sialylated oligosaccharides have been identified in humans as a recurring constituent of the upper respiratory epithelial glycocalix [29]. For instance, tetrasaccharide **30** has been reported to bind to haemagglutinins isolated from different H1N1 human viral strains, and was chosen as a target to showcase the solid-phase automated synthesis of α-(2→6) sialosides (Scheme 6).

The synthesis of tetrasaccharide **30** started with the glycosylation of linker **17** by using galactose building block **28** under standard conditions for the activation of thioglycosides, followed by Fmoc removal and a glycosylation with building block **18**. The solid-phase bound disaccharide was further elongated, following removal of the temporary protecting group, by reaction with building block **5**. For this reaction, we applied the reaction conditions optimized for building block **4** without further optimization. Thus, standard TCA reduction, cleavage from the support, saponification and isolation afforded semiprotected tetrasaccharide **29** in 16% overall yield. As shown by LC–MS analysis (see Supporting Information File 1) the crude mixture contains the desired tetrasaccharide, but also some deletion sequences that can be attributed to the non-optimized conditions used for the synthesis. Moreover, building blocks **18** and **28** have been observed to be a non-ideal donor–acceptor pair (unpublished results). Nevertheless, simple reverse-phase HPLC was sufficient to isolate semiprotected tetrasaccharide **29** in milligram quantities. To complete the synthesis, hydrogenolysis was performed to give final tetrasaccharide **30** in 51% yield. The above example shows that the platform can provide access to target oligosaccharides by using generalized coupling protocols even when conditions are not optimized.

#### Formation of biotinylated probes

**Scheme 7:**
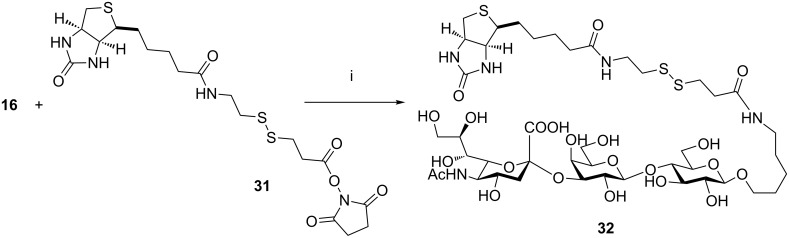
Reagents and conditions: (i) 10% DMF in PBS buffer pH 7.5, overnight, 80%.

The syntheses of glycans **27** and **30** showed the efficiency of sialyl building blocks **4** and **5** in combination with the automated solid-phase platform for the rapid and reliable access to complex sialosides. Our synthetic strategy makes use of linker **17**, which incorporates an amino spacer for conjugation into the final oligosaccharide. In this way, the synthetic sialosides can be easily conjugated to probes for biological evaluation or labelled for instance with UV-active tags. Biotinylation is a typical example of a commonly employed labelling technique [30] and has been extensively used for instance as a functionalization technique for antigens in antibody selection by phage-display methods [31]. Thus, trisaccharide **16** (Scheme 7) was reacted with biotin derivative **31** in PBS buffer to afford compound **32** in 80% yield after gel filtration.

## Conclusion

The synthesis of sialosides is important to create tools for glycobiology. The work presented here demonstrates that several important sialylated oligosaccharides can be accessed by using a standardized automated approach. Two sialic acid containing disaccharide building blocks containing either α-(2→3) or α-(2→6) galactose linkages were obtained in high overall yields from readily accessible starting materials. In combination with a fully automated synthesizer, the disaccharide building blocks have been exploited for the solid-phase synthesis of several oligosaccharides ready for biological evaluation. This work represents the first full account of an automated solid-phase synthesis of sialosides.

## Supporting Information

File 1 Experimental procedure and characterization data for new compounds.

File 2 ^1^H and ^13^C NMR spectra for new compounds.
