# Supplementary material for: Automated synthesis of sialylated oligosaccharides
Source: Beilstein J Org Chem. 2012 Sep 21;8:1601–9. doi: 10.3762/bjoc.8.183 (PMC3510992; doi:10.3762/bjoc.8.183)
Supplement: File 1 — Experimental procedure and characterization data for new compounds. [file Beilstein_J_Org_Chem-08-1601-s001.pdf]

Supporting Information

for

Automated synthesis of sialylated oligosaccharides

Davide Esposito[1,2], Mattan Hurevich[1,2], Bastien Castagner[3], Cheng-Chung Wang[4] and Peter H. Seeberger[1,2*]

Address: [1]Max-Planck-Institute of Colloids and Interfaces, Department of Biomolecular Systems, Am Mühlenberg 1, 14476 Potsdam, Germany, [2]Freie Universität Berlin, Institute of Chemistry and Biochemistry, Arnimallee 22, 14195 Berlin, Germany, [3]Institute of Pharmaceutical Sciences, Swiss Federal Institute of Technology (ETH) Zurich, 8093 Zurich, Switzerland and [4]Institute of Chemistry, Academia Sinica, Taipei, 11529, Taiwan

Email: Peter H. Seeberger - Peter.Seeberger@mpikg.mpg.de

[*]Corresponding author

Experimental procedures and characterization data for new compounds

Table of contents

General material and methods S2

Synthesis and characterization data of new compounds S3

References S13

General materials and methods. All chemicals used were reagent grade and used as supplied. All reactions were performed in oven-dried glassware under an inert atmosphere unless noted otherwise. Reagent grade *N*,*N*-dimethylformamide (DMF) was dried over activated molecular sieves prior to use. Dichloromethane (CH_2Cl_2), toluene and tetrahydrofuran (THF) were purified by a Cycle-Tainer Solvent Delivery System unless noted otherwise. All solvents used on the automated synthesizer were extra dry grade without molecular sieves, purchased from Acros in sure seal bottles except DCM and THF, which were dried by using a dry still. Analytical thin-layer chromatography (TLC) was performed on Merck silica gel 60 F_{254} plates (0.25 mm). Compounds were visualized by UV irradiation or dipping the plate in a cerium sulfate-ammonium molybdate (CAM) solution. Flash column chromatography was carried out by using forced flow of the indicated solvent on Fluka Kieselgel 60 (230–400 mesh). Purification by reverse phase HPLC was performed by using Agilent 1200 series equipped with a Macherey-Nagel Nucleodur Pyramid C-18 column (length 250 mm, 40 mm i.d., flow 10 mL/min) unless noted otherwise. ^{1}H, ^{13}C NMR spectra were recorded on a Varian Mercury 300 (300 MHz), Varian 400-MR (400 MHz), Varian 600-MR (600 MHz), Bruker ECX (400 MHz), Bruker DRX500 (500 MHz), or Bruker DRX700 (700 MHz) spectrometer in $CDCl_3$ by using the solvent residual peak chemical shift as the internal standard ($CDCl_3$: 7.26 ppm ^{1}H, 77.0 ppm ^{13}C) unless otherwise stated. NMR chemical shifts (δ) are reported in ppm and coupling constants (*J*) are reported in Hz. High-resolution mass spectral (HRMS) analyses were performed by the MS-service at the MS-service at Department of Organic Chemistry at Free University Berlin. High-resolution MALDI and ESI mass spectra were run on IonSpec Ultima instruments. IR spectra were recorded on a Perkin-Elmer 1600 FTIR spectrometer. Optical rotations were measured by using a Perkin-Elmer 241 and Unipol L1000 polarimeter.

Synthesis and characterization data of new compounds

(*N*-acetyl-4,7,8,9-tetra-*O*-acetyl-1-methyl-α-neuraminosyl)-(2→6)-α-2-*O*-acetyl-3,4-di-*O*-benzyl-D-galactopyranosyl *N*-phenyl-trifluoroacetimidate (5)

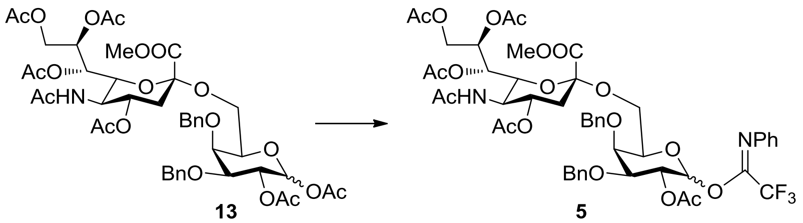

To a solution of **13** [S1] (240 mg, 261 μmol) in DMF (4 mL), hydrazine acetate (52 mg, 575 μmol) was added. After stirring for 15 h at room temperature under argon atmosphere, the mixture was diluted with EtOAc and washed twice with a 10% citric acid solution (100 mL). The aqueous phase was extracted with EtOAc, and the combined organic layers were washed with brine, dried over $MgSO_4$, filtered and concentrated. Purification by flash column silica gel chromatography (cyclohexane/EtOAc = 5:1 to 0:1) gave the corresponding hemiacetal (205 mg, 234 μmol, 90%). The latter was dissolved in dichloromethane (5 mL) and Cs_2CO_3 (152 mg, 468 μmol) and $CF_3C(NPh)Cl$ (145 mg, 700 μmol) were added. After stirring overnight at room temperature under an argon atmosphere, the mixture was filtered through celite and concentrated. Silica column chromatography (cyclohexane/EtOAc = 7:3 to 0:1 + 0.1% Et_3N) afforded the desired product **5** as a mixture of anomers (210 mg, 201 μmol, 86%). ^{1}H NMR (600 MHz, $CDCl_3$) δ 7.40–7.15 (m, 12H), 7.02 (t, *J* = 7.1 Hz, 1H), 6.78 (d, *J* = 6.6 Hz, 2H), 5.57–5.43 (m, 1H), 5.34–5.27 (m, 1H), 5.27–5.21 (m, 1H), 5.21–5.14 (m, 1H), 4.88 (d, *J* = 11.4 Hz, 1H), 4.82–4.74 (m, 1H), 4.64–4.53 (m, 2H), 4.47 (d, *J* = 12.1 Hz, 1H), 4.24–4.16 (m, 1H), 4.07–3.96 (m, 3H), 3.93–3.84 (m, 2H), 3.71–3.55 (m, 1H), 3.58–3.52 (m, 4H), 3.47 (m, 1H), 2.51 (dd, *J* = 12.9, 4.6 Hz, 1H), 2.05 (s, 3H), 2.02 (s, 3H), 1.97 (s, 3H), 1.95 (s, 3H), 1.91 (s, 3H), 1.86 (dd, *J* = 12.7, 4.8 Hz, 1H), 1.81 (s, 3H); ^{13}C NMR (151 MHz, $CDCl_3$) δ 169.88, 169.61, 169.27, 169.00, 168.76, 168.12, 166.79, 142.39, 137.57, 137.35, 136.82, 136.68, 134.30, 128.31,

127.70, 127.53, 127.44, 127.19, 126.83, 126.71, 126.62, 126.46, 126.41, 125.23, 123.26, 119.51, 118.24, 97.61, 78.79, 73.34, 72.97, 71.58, 71.15, 71.11, 69.22, 68.00, 67.55, 66.30, 61.55, 61.40, 61.23, 51.79, 48.38, 36.82, 36.76, 22.17, 20.00, 19.80, 19.74, 19.66; ESI–MS *m/z:* [M − OCNPh(CF_3)]$^+$ calcd for $C_{42}H_{52}NO_{18}$: 858.32; found: 858.20; IR (thin film) ν: 2992, 2853, 1741, 1455, 1368, 1214.

_N_-(Benzyl)-_N_-benzyloxycarbonyl-5-aminopentyl 2-acetyl-3,6-di-_O_-benzyl-β-D-glucopyranoside (14)

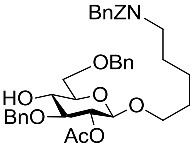

14

To a cold solution (0 °C) of ethyl 2-acetyl-3,6-di-*O*-benzyl-4-*O*-fluorenylmethoxycarbonyl-thio-α-D-glucopyranoside [S2] (190 mg, 0.241 mmol) and *N*-benzyl-*N*-benzyloxycarbonyl-5-aminopentan-1-ol [S3] (134 mg, 0.327 mmol) in DCM (1.5 mL) under an atmosphere of argon and in the presence of 4Å AW-MS (acid-washed molecular sieves, 5 rods), NIS (65 mg, 0.290 mmol) and TfOH (4.29 μL, 0.048 mmol) were added. The reaction was stirred for 2 hours at 0 °C, then quenched with triethylamine (100 μL). After warming up to room temperature, the reaction mixture was stirred for one hour at room temperature, then filtered over celite and concentrated in vacuum. Purification with flash column silica gel chromatography (hexane/ethylacetate = 9:1 to 2:1) gave compound **14** (137 mg, 0.192 mmol, 80% yield). **14:** $[\alpha]_D^{20}$ −12.10 (*c* 1, $CHCl_3$); ^{1}H NMR (600 MHz, $CDCl_3$) δ 7.40–7.02 (m, 20H), 5.09 (d, *J* = 17.1 Hz, 2H), 4.87 (t, *J* = 8.7 Hz, 1H), 4.75–4.56 (m, 2H), 4.50 (dd, *J* = 27.6, 12.0 Hz, 2H), 4.40 (d, *J* = 11.5 Hz, 2H), 4.25 (dd, *J* = 19.6, 7.5 Hz, 1H), 3.75–3.58 (m, 4H), 3.46–3.38 (m, 2H), 3.34–3.24 (m, 1H), 3.12 (d, *J* = 41.2 Hz, 2H), 1.89 (d, *J* = 18.3 Hz, 3H), 1.51–1.35 (m, 4H), 1.24–1.09 (m, 2H); ^{13}C NMR (151 MHz, $CDCl_3$) δ 169.48, 156.18, 156.73, 136.85, 138.46, 138.04, 137.85, 128.65, 128.62, 128.59, 127.95, 127.94, 127.86, 101.13, 82.50, 74.42, 74.11, 73.88, 72.86,

72.48, 70.65, 67.28, 63.34, 50.50, 50.19, 47.06, 46.13, 29.29, 27.81, 27.37, 23.26, 21.02; HRMS–ESI: *m/z* [M + Na]$^+$ calcd for $C_{42}H_{49}NNaO_9$: 734.3305; found: 734.3274; IR (thin film) ν: 3457, 2910, 2323, 1746, 1691, 1420, 1367, 1240, 1059.

***N*-(Benzyl)-*N*-benzyloxycarbonyl-5-aminopentyl (5-acetamido-4,7,8,9-tetra-*O*-acetyl-3,5-dideoxy-1-methyl-D-*glycero*-α-D-*galacto*-non-2-ulopyranosylonic acid)-(2→3)-2-*O*-acetyl-4,6-di-*O*-benzyl-β-D-galactopyranosyl-(1→4)-2-*O*-acetyl-3,6-di-*O*-benzyl-β-D-glucopyranoside (15)**

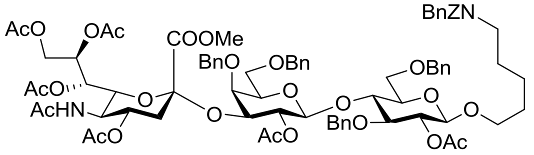

15

To a solution of sialyl galactose donor **4** (220 mg, 0.210 mmol) and glucose **14** (93 mg, 0.131 mmol) in CH_2Cl_2 (2.0 mL) was added TMSOTf (4.7 μL, 0.026 mmol) at 0 °C under an Ar atmosphere, in the presence of 4Å AW-MS (acid-washed molecular sieves, 5 rods). After stirring for 1.5 h at 0 °C, the mixture was neutralized with a few drops of Et_3N and then concentrated. Purification with flash column silica gel chromatography (hexane/ethylacetate = 2:1 to 1:1) gave trisaccharide **15** (120 mg, 0.16 mmol, 80%); ^{1}H NMR (600 MHz, $CDCl_3$) δ 7.40–7.10 (m, 30H), 5.59 (ddd, *J* = 8.8, 6.2, 2.7 Hz, 1H), 5.36 (dd, *J* = 8.6, 2.4 Hz, 1H), 5.26 (dd, *J* = 10.1, 7.8 Hz, 1H), 5.21–5.12 (m, 3H), 4.95 (d, *J* = 11.6 Hz, 1H), 4.92 (t, 1H), 4.87–4.78 (m, 3H), 4.62 (q, *J* = 12.1 Hz, 2H), 4.56 (d, *J* = 11.6 Hz, 1H), 4.47 (d, *J* = 10.8 Hz, 2H), 4.40–4.33 (m, 2H), 4.30–4.25 (m, 3H), 4.16 (d, *J* = 11.7 Hz, 1H), 4.06 (dd, *J* = 20.8, 10.4 Hz, 1H), 3.99 (dd, *J* = 12.4, 6.2 Hz, 1H), 3.91–3.84 (m, 2H), 3.82 (dd, *J* = 10.8, 2.4 Hz, 1H), 3.70 (dd, *J* = 11.0, 5.7 Hz, 1H), 3.67 (s, 3H), 3.60–3.54 (m, 2H), 3.47 (t, *J* = 8.7 Hz, 2H), 3.43 (d, *J* = 2.4 Hz, 1H), 3.37 (m, 1H), 3.25 (dd, *J* = 9.2, 5.2 Hz, 2H), 3.16 (s, 1H), 2.64 (dd, *J* = 12.7, 4.5 Hz, 1H), 2.17 (s, 3H), 2.11 (s, 3H), 2.05 (s, 3H), 2.02 (s, 3H), 1.97 (s, 3H), 1.94–1.88 (m, 4H), 1.86 (s, 3H), 1.51–1.35 (m, 4H), 1.24–1.09 (m,

2H); ^{13}C NMR (151 MHz, $CDCl_3$) δ 171.15, 170.89, 170.44, 170.34, 169.91, 169.68, 169.65, 169.37, 168.07, 156.68, 156.13, 139.01, 138.82, 138.57, 138.22, 137.87, 136.75, 128.50, 128.42, 128.23, 128.21, 128.09, 128.00, 127.93, 127.80, 127.52, 127.45, 127.35, 127.29, 127.21, 126.96, 100.78, 100.73, 97.61, 81.25, 77.12, 75.46, 75.01, 74.67, 74.62, 74.58, 73.22, 73.03, 72.81, 72.46, 72.27, 71.09, 69.24, 69.09, 68.78, 68.04, 67.65, 67.37, 67.13, 62.32, 60.39, 52.83, 50.48, 50.14, 49.18, 47.04, 46.15, 37.63, 29.14, 27.82, 27.38, 23.16, 23.11, 21.26, 21.05, 20.84, 20.79, 20.72, 20.70, 14.19; ESI–MS *m*/*z*: $[M + NH_4]^+$ calcd for $C_{84}H_{104}N_3O_{27}$: 1586.69; found: 1586.1, 792.5 $[M + H + NH_4]^{2+}$.

Aminopentyl (5-acetamido-3,5-dideoxy-D-*glycero*-α-D-*galacto*-non-2-ulopyranosylonic acid)-(2→3)-β-D-galactopyranosyl-(1→4)-β-D-glucopyranoside (16)

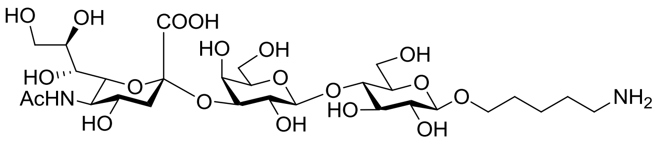

16

Compound **15** (150 mg, 0.076 mmol) was dissolved in methanol (1 mL) and a few drops of dichloromethane. A solution of Na in methanol (250 μL, 0.015 mmol) was added and the reaction was stirred overnight at room temperature. The reaction was quenched with amberlite IR-120, filtered over celite and concentrated. The crude residue was dissolved in methanol/water (7 mL, 8:2) and 1 M aqueous KOH (300 μL) was added. The reaction mixture was stirred at 60 °C overnight, quenched with amberlite IR-120, filtered over celite and concentrated under reduced pressure to give 84 mg (0.064 mmol) of the intermediate compound in which all esters have been hydrolysed. Finally, a portion of the crude residue (70 mg, 0.053 mmol) was dissolved in methanol/water (7.5 mL, 8:2) and acetic acid (120 μL) was added to the solution

followed by Pd/C (60 mg). The mixture was sonicated under argon flow for 20 min, then under hydrogen flow for an additional 20 min and finally stirred under an atmosphere of hydrogen for 36 h. The catalyst was removed by filtration through a pad of celite and the filtrate was concentrated and lyophilized. The crude residue was purified by preparative HPLC using a 0 to 20% gradient (CH_3CN in water + 0.1% TFA, Pyramid nucleodur C-18 column) over 35 min to afford **16** (34 mg) in 76% yield over three steps. The purity of **16** was assessed by analytical HPLC by using a 95 to 5% gradient (CH_3CN in water, TSKgel amide-80 TOSOH) as shown in Figure SI-1. ^{1}H NMR (600 MHz, D_2O) δ 4.53 (dd, *J* = 7.9, 25.1 Hz, 2H), 4.14 (dd, *J* = 2.9, 9.8 Hz, 1H), 4.03–3.93 (m, 3H), 3.92–3.81 (m, 4H), 3.80–3.64 (m, 9H), 3.63–3.55 (m, 3H), 3.32 (t, *J* = 12.0 Hz, 1H), 3.03 (t, *J* = 7.4 Hz, 2H), 2.78 (dd, *J* = 4.5, 12.4 Hz, 1H), 2.05 (s, 3H), 1.83 (t, *J* = 12.2 Hz, 1H), 1.75–1.65 (m, 4H), 1.51–1.44 (m, 2H); ^{13}C NMR (151 MHz, D_2O) δ 174.8, 173.4, 102.6, 102.0, 99.6, 78.3, 75.5, 75.1, 74.7, 74.4, 72.9, 72.8, 71.6, 70.0, 69.3, 68.2, 68.1, 67.4, 62.6, 61.0, 60.0, 51.6, 39.5, 39.3, 28.1, 26.3, 22.00, 21.98; MALDI–MS *m*/*z*: [M + Na^+] calcd for $C_{28}H_{50}N_2NaO_{19}$: 741.290, found: 741.383.

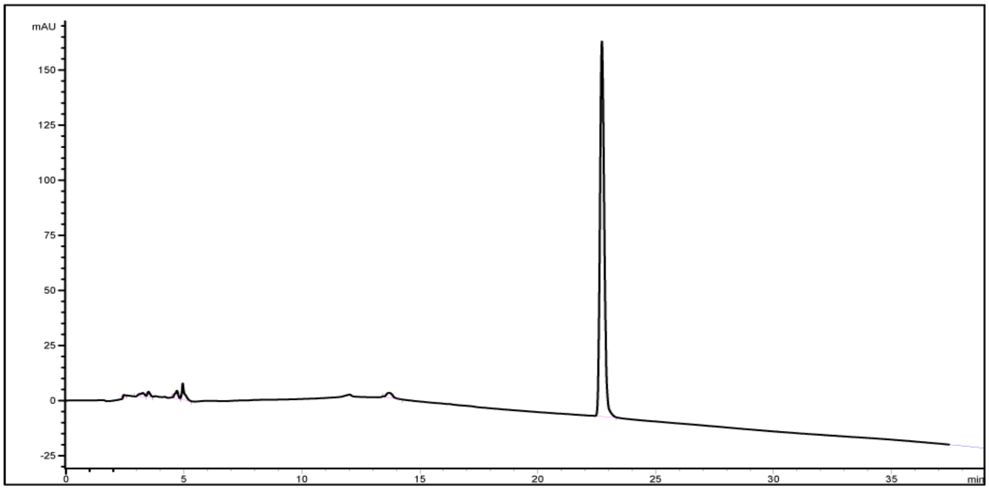

Figure SI-1: LC–MS of **16**. Conditions: 95 to 5% A in B; A = CH_3CN, B = water, over 30 min.

5-(benzyl(((4-(2-carboxyethyl)benzyl)oxy)carbonyl)amino)pentyl (5-acetamido-3,5-dideoxy-D-*glycero*-α-D-*galacto*-non-2-ulopyranosylonic acid)-(2→6)-3,4-di-*O*-benzyl-β-D-galactopyranosyl-(1→4)-2-acetamido-2-deoxy-3,6-di-*O*-benzyl-β-D-glucopyranosyl-(1→3)-4,6-di-*O*-benzyl-β-D-galactopyranoside (29)

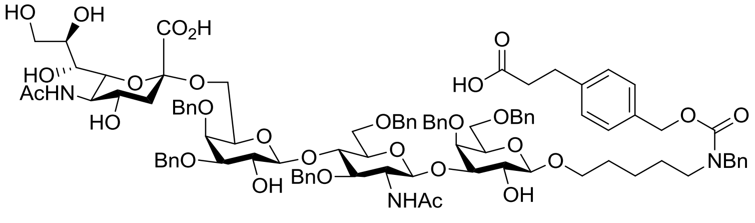

29

The following module sequences (modules have been defined in [S4]) were performed on 25 µmol scale with respect to resin **17**:

1) Thioglycoside double coupling–Fmoc deprotection with building block **28** [S4]
2) Thioglycoside double coupling–Fmoc deprotection with building block **18** [S4]
3) Glycosyl Imidate double coupling (0 °C) with building block **5**
4) TCA Reduction by treatment with 10 equiv of Bu_3SnH/AIBN
5) Linker Cleavage by treatment with 10 equiv NaOMe

The eluent from the synthesizer was neutralized with acidic Amberlite IR-120 resin and concentrated under reduced pressure. The crude residue was dissolved in methanol (1.6 mL), water (0.4 mL), and THF (0.5 mL), and aqueous KOH (1N, 300 µL, ca. 14 equiv) was added. The reaction mixture was stirred at 60 °C overnight, quenched with amberlite IR-120 resin, filtered through a pad of celite and concentrated. The crude residue was purified by preparative HPLC using a 40 to 95% gradient (CH_3CN in water + 0.1% formic acid) over 40 min to afford the desired compound **29** (7.12 mg, 4 µmol) in 16% yield from the resin. The purity of **29** was assessed by analytical HPLC using a 30 to 95% gradient

(CH_3CN in water + 0.1% formic acid) over 40 minutes, as shown in Figure SI-2 b. ^{1}H NMR (600 MHz, CD_3OD) δ 7.37 (d, *J* = 7.1 Hz, 2H), 7.28–7.12 (m, 37H), 5.03 (d, *J* = 12.6 Hz, 2H), 4.86 (dd, *J* = 20.1, 11.3 Hz, 3H), 4.76–4.73 (m, 1H), 4.69 (m, 2H), 4.58–4.52 (m, 2H), 4.46 (m, 3H), 4.38 (m, 4H), 4.30 (d, *J* = 11.9 Hz, 1H), 4.11–4.09 (m, 1H), 3.98–-3.83 (m, 5H), 3.83–3.77 (m, 3H), 3.73–3.69 (m, 4H), 3.67–3.61 (m, 3H), 3.61–3.53 (m, 6H), 3.49 (d, *J* = 9.7 Hz, 2H), 3.44 (m, 3H), 3.37–3.32 (m, 3H), 3.18–3.13 (m, 2H), 2.84 (t, *J* = 7.5 Hz, 2H), 2.65–2.63 (m, 1H), 2.55 (d, *J* = 7.6 Hz, 2H), 1.95 (s, 3H), 1.85 (s, 3H), 1.67 (t, *J* = 12.2 Hz, 1H), 1.51–1.38 (m, 4H), 1.27–1.23 (m, 2H); ^{13}C NMR (151 MHz, CD_3OD) δ 175.3, 175.0, 173.5, 171.6, 140.35, 140.33, 140.0, 139.62, 139.51, 129.73, 129.64, 129.58, 129.52, 129.46, 129.38, 129.34, 129.23, 129.21, 129.18, 129.14, 129.11, 129.08, 128.99, 128.90, 128.76, 128.72, 128.65, 128.46, 128.43, 128.40, 128.37, 105.0, 104.29, 104.28, 100.0, 84.0, 83.7, 83.5, 81.9, 77.73, 77.70, 76.8, 76.3, 75.91, 75.79, 75.54, 75.47, 75.2, 74.66, 74.56, 74.54, 74.46, 74.42, 74.37, 74.28, 73.65, 73.58, 72.93, 72.88, 72.49, 72.41, 72.0, 70.7, 70.3, 70.02, 69.96, 69.86, 68.8, 68.3, 67.6, 64.3, 63.1, 56.9, 53.85, 53.77, 51.5, 51.3, 49.6, 47.5, 41.7, 36.5, 31.6, 30.3, 29.9, 28.8, 28.4, 24.2, 23.2, 22.6; HRMS–ESI *m*/*z*: $[M – H]^–$ calcd for $C_{96}H_{114}N_3O_{28}$: 1756.7589, found: 1756.7595.

a)

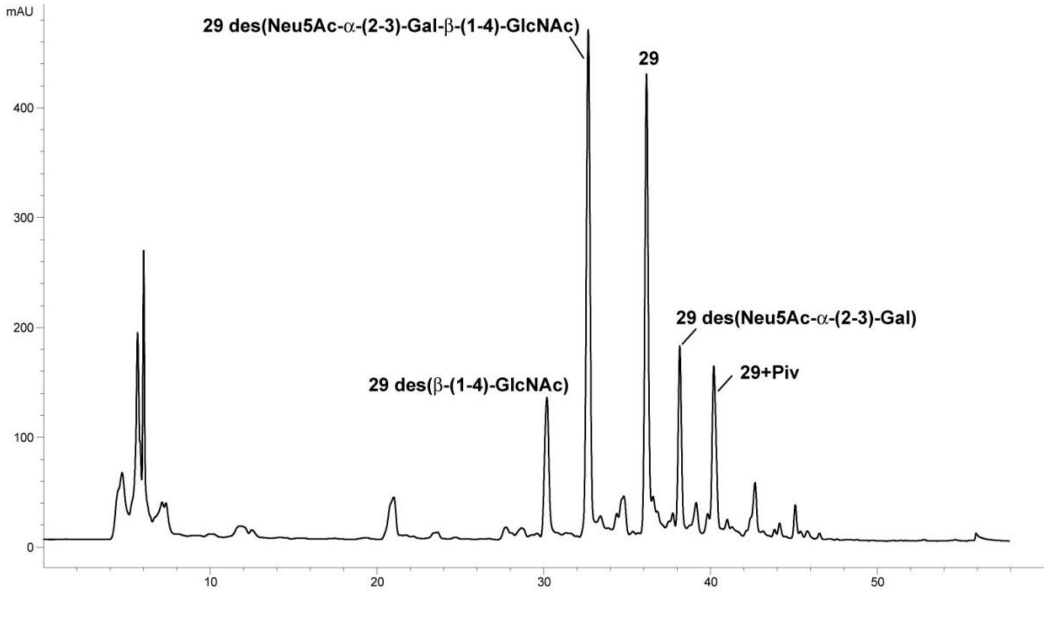

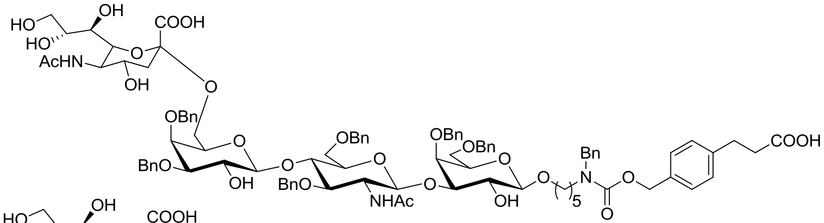

29

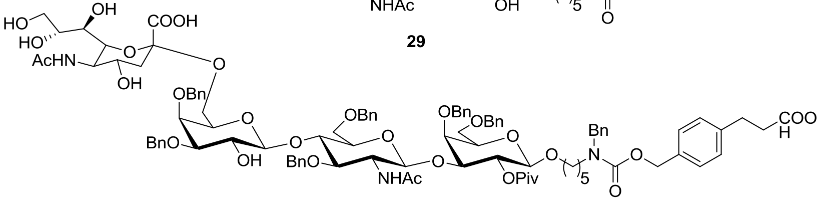

29+Piv

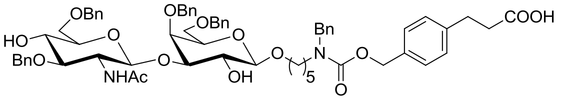

29 desNeu5Ac-α–(2-3)-Gal

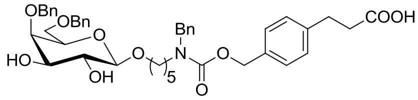

29 des(Neu5Ac-α–(2-3)-Gal-β-(1-4)-GlcNAc)

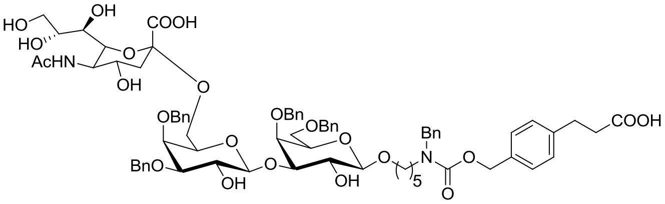

29 des(β-(1-4)-GlcNAc)

b)

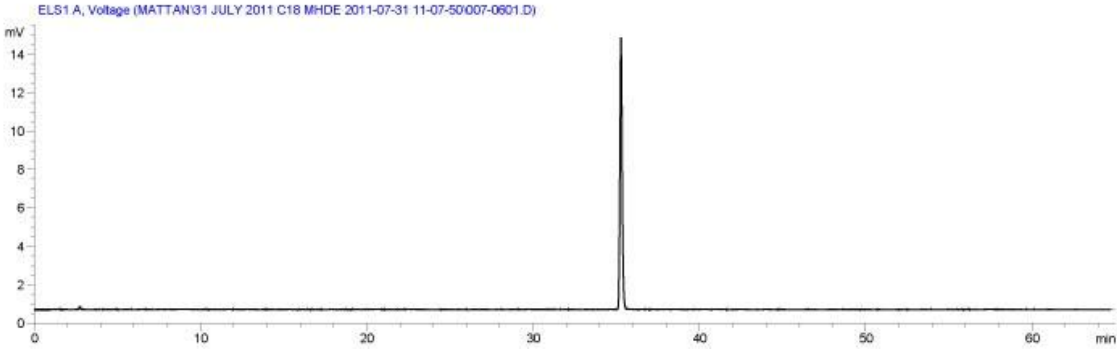

Figure SI-2: (a) LC–MS trace of crude **29**, conditions: 40 to 95% A in B over 40 min; A = CH_3CN + 0.1% formic acid, B = water + 0.1% formic acid. (b) Pure **29**, conditions: 30 to 95% A in B over 40 min; A = CH_3CN + 0.1% formic acid, B = water + 0.1% formic acid.

Aminopentyl (5-acetamido-3,5-dideoxy-D-*glycero*-α-D-*galacto*-non-2-ulopyranosylonic acid)-(2→6)-β-D-galactopyranosyl-(1→4)-2-acetamido-2-deoxy-β-D-glucopyranosyl-(1→3)-β-D-galactopyranoside (30)

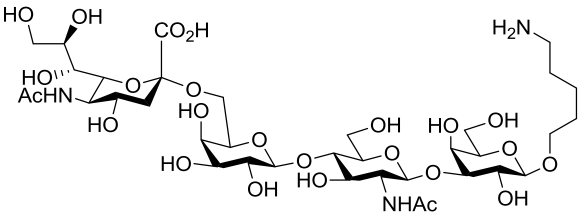

30

To a suspension of **29** (7.2 mg, 4 μmol) in methanol/water/ethyl acetate (1 mL, 2:2:1), acetic acid (30 μL) was added followed by Pd/C (10 mg). The mixture was sonicated under argon flow for 20 min, then under hydrogen flow for an additional 20 min and finally stirred under an atmosphere of hydrogen for 48 h. The catalyst was removed by filtration through a pad of celite and the filtrate was concentrated and lyophilized. The crude residue was purified by HPLC using a 0.1 to 15% gradient (CH_3CN in water + 0.1% FA, Waters X-bridge C-18 column) over 10 min to afford the desired compound **30** (1.9 mg, 2 μmol) in 51% yield. ^1H NMR (600 MHz, D_2O) δ 4.34 (d, *J* = 8.09, 1H), 4.26 (d, *J* = 8.1 Hz, 1H), 4.03 (d, *J* = 3.48, 1H), 3.87 (t, *J* = 9.7 Hz, 1H), 3.82–3.80 (m, 3H), 3.74 (m, 3H), 3.69 (m, 2H), 3.67 (m, 2H), 3.63 (m, 2H), 3.57–3.52 (m, 8H), 3.51–3.46 (m, 2H), 3.42 (m, 4H), 2.88 (t, *J* = 7.5 Hz, 2H), 2.55 (dd, *J* = 12.4, 4.7 Hz, 1H), 1.93 (s, 3H), 1.90 (s, 3H), 1.59–1.53 (m, 5H), 1.34 (m, 2H); ^{13}C NMR extracted from HSQC (151 MHz, D_2O) δ 102.33, 103.32, 102.69, 82.23, 80.41, 74.51, 74,16, 73.60, 72.50, 72.35, 72.12, 71.59, 70.63, 69.83, 69.62, 68.25, 68.14, 63.25, 62.67, 62.61, 62.53, 60.79, 60.08, 54.90, 53.96, 51.81, 39.95, 39.22, 28.05, 26.28, 22.17, 21.93, 21.90; HRMS–ESI *m/z*: $[M + K]^+$ calcd for $C_{36}H_{63}KN_3O_{24}$: 960,3439, found: 961.4016.

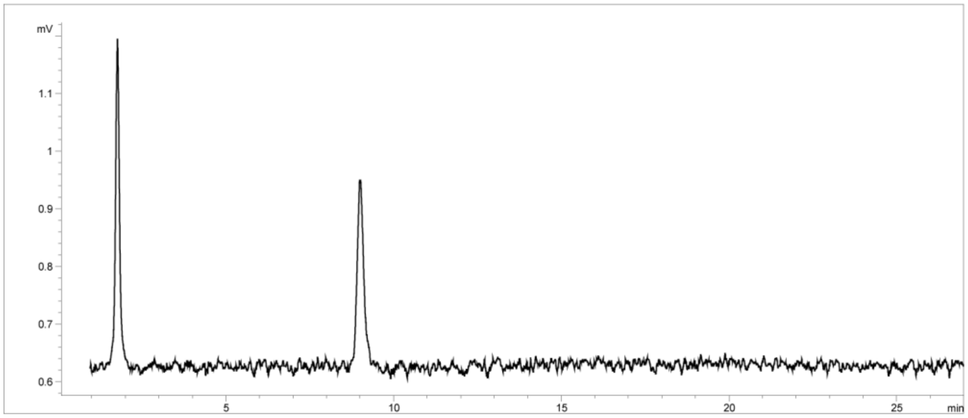

Figure SI-3: LC–MS trace of **30**. Conditions: 0.1 to 15% A in B over 10 min; A = CH_3CN, B = water (column Waters X-bridge C-18, particle size 5 μm, 4.6 × 150 mm).

5-(3-((2-(Biotinamido)ethyl)disulfanyl)propanamido)pentyl (5-acetamido-3,5-dideoxy-D-*glycero*-α-D-*galacto*-non-2-ulopyranosylonic acid)-(2→3)-β-D-galactopyranosyl-(1→4)-β-D-glucopyranoside 32.

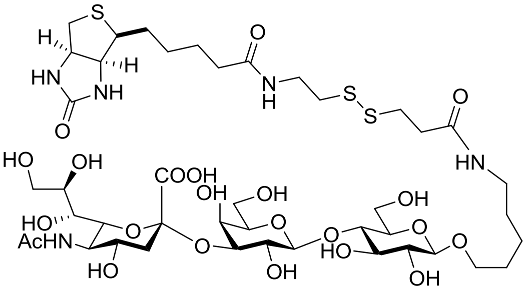

32

Compound **16** (1.5 mg, 2.1 μmol) was dissolved in PBS buffer (pH 7.5, 400 μL) in an eppendorf tube. A DMF solution of NHS-SS-Biotin (40 μL, 0.08 M) was added and the reaction was agitated overnight at rt. The solution was lyophilized and the crude residue was purified by size-exclusion chromatography (LH-20 Sephadex, water) to afford the title compound **32** in 80% yield (2 mg, 1.8 μmol). ^{1}H NMR (600 MHz, D_2O) δ 4.62 (dd, *J* = 7.6, 5.0 Hz, 1H), 4.54 (d, *J* = 7.8 Hz, 1H), 4.49 (d, *J* = 8.0 Hz, 1H), 4.45 (t, *J* = 3.9 Hz, 1H), 4.12 (dd, *J* = 9.8, 3.0 Hz, 1H), 4.01–3.97 (m, 2H), 3.94–3.89 (m, 2H), 3.88–3.81 (m, 2H), 3.77 (dd, *J* =

13.4, 10.0 Hz, 2H), 3.73–3.60 (m, 8H), 3.54 (t, J = 5.2 Hz, 2H), 3.35 (dt, J = 9.4, 4.8 Hz, 1H), 3.31 (t, J = 8.5 Hz, 1H), 3.22 (t, J = 6.7 Hz, 2H), 3.00 (m, 4H), 2.90 (t, J = 6.1 Hz, 2H), 2.78 (m, 2H), 2.67 (t, J = 6.6 Hz, 2H), 2.29 (t, J = 7.1 Hz, 2H), 2.04 (s, 3H), 1.81 (t, J = 12.1 Hz, 1H), 1.78–1.64 (m, 6H), 1.62–1.53 (m, 4H), 1.48–1.39 (m, 4H); ^{13}C NMR (151 MHz, D_2O) δ 176.9, 174.9, 173.91, 173.80, 102.6, 102.0, 99.7, 78.3, 75.4, 75.1, 74.7, 74.4, 72.8, 71.7, 70.3, 69.3, 68.3, 68.0, 67.4, 62.5, 62.0, 61.2, 61.0, 60.18, 55.4, 51.6, 39.7, 39.32, 39.20, 37.8, 36.8, 35.4, 35.0, 33.4, 30.6, 28.3, 27.91, 27.78, 27.61, 26.4, 25.1, 22.4, 22.0; ESI–MS *m/z*: $[M - H]^-$ calcd for $C_{43}H_{72}N_5O_{22}S_3$: 1107.39, found: 1106.3, 1130.2 $[M + Na]^+$.

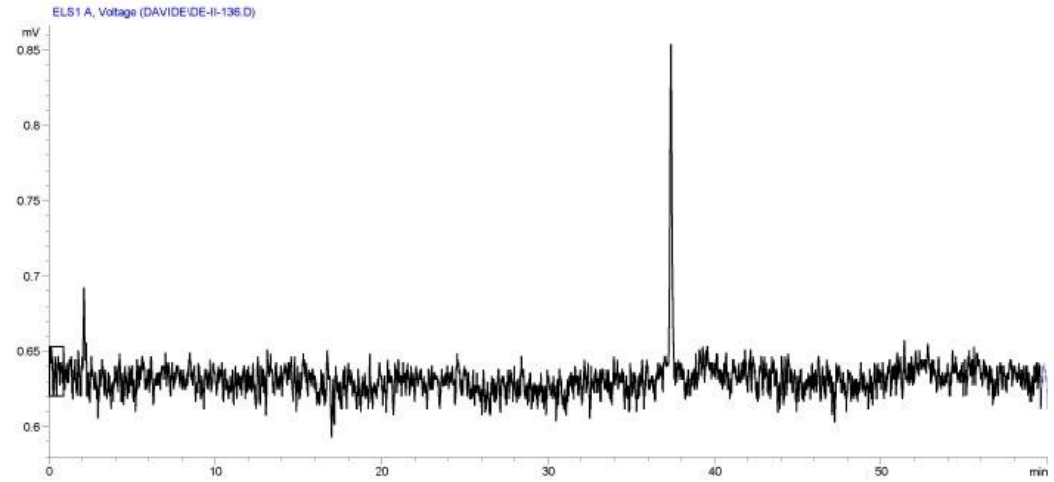

Figure SI-4: LC–MS trace of **32**. Conditions: 0 to 50% A in B over 30 min; A = CH_3CN + 0.1% FA, B = water + 0.1% FA (column Nucleodur Pyramid C-18).

References

S1. Wang, P.; Zhu, J.; Yuan, Y.; Danishefsky, S. J. *J. Am. Chem. Soc.* **2009**, *131*, 16669–16671.

S2. Wang, C. C.; Lee, J. C.; Luo, S. Y.; Kulkarni, S. S.; Huang, Y. W.; Lee, C. C.; Chang, K. L.; Hung, S. C. *Nature* **2007**, *446*, 896–899.

S3. T. K.-K. Mong, H.-K. Lee, S. G. Duron, C.-H. Wong, *PNAS* **2003**, *100*, 797–802.

S4. Kröck, L.; Esposito, D.; Castagner, B.; Wang, C.-C.; Bindschädler, P.; Seeberger, P. H. *Chem. Sci.* **2012**, *3*, 1617–1622.
