# Supplementary material for: Automated synthesis of sialylated oligosaccharides
Source: Beilstein J Org Chem. 2012 Sep 21;8:1601–9. doi: 10.3762/bjoc.8.183 (PMC3510992; doi:10.3762/bjoc.8.183)
Supplement: File 2 — 1H and 13C NMR spectra for new compounds. [file Beilstein_J_Org_Chem-08-1601-s002.pdf]

Supporting Information

for

Automated synthesis of sialylated oligosaccharides

Davide Esposito[1,2], Mattan Hurevich[1,2], Bastien Castagner[3], Cheng-Chung Wang[4] and Peter H. Seeberger[1,2*]

Address: [1]Max-Planck-Institute of Colloids and Interfaces, Department of Biomolecular Systems, Am Mühlenberg 1, 14476 Potsdam, Germany, [2]Freie Universität Berlin, Institute of Chemistry and Biochemistry, Arnimallee 22, 14195 Berlin, Germany, [3]Institute of Pharmaceutical Sciences, Swiss Federal Institute of Technology (ETH) Zurich, 8093 Zurich, Switzerland and [4]Institute of Chemistry, Academia Sinica, Taipei, 11529, Taiwan

Email: Peter H. Seeberger - Peter.Seeberger@mpikg.mpg.de

[*]Corresponding author

^{1}H and ^{13}C NMR spectra for new compounds

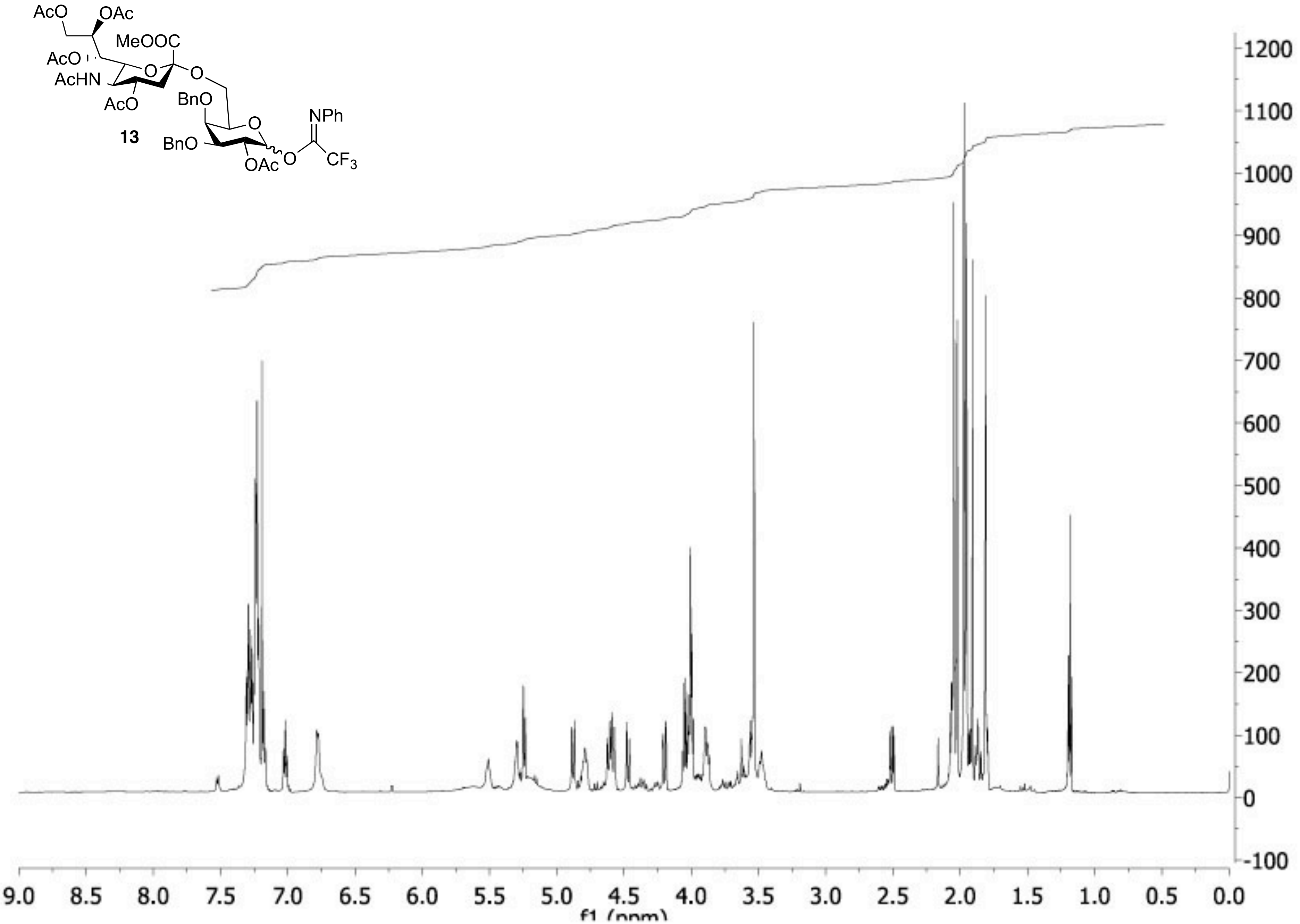
AcO
OAc
MeOOC
AcO
AcHN
O
O
AcO
BnO
O
NPh
13
BnO
OAc
O
CF3
f1 (ppm)

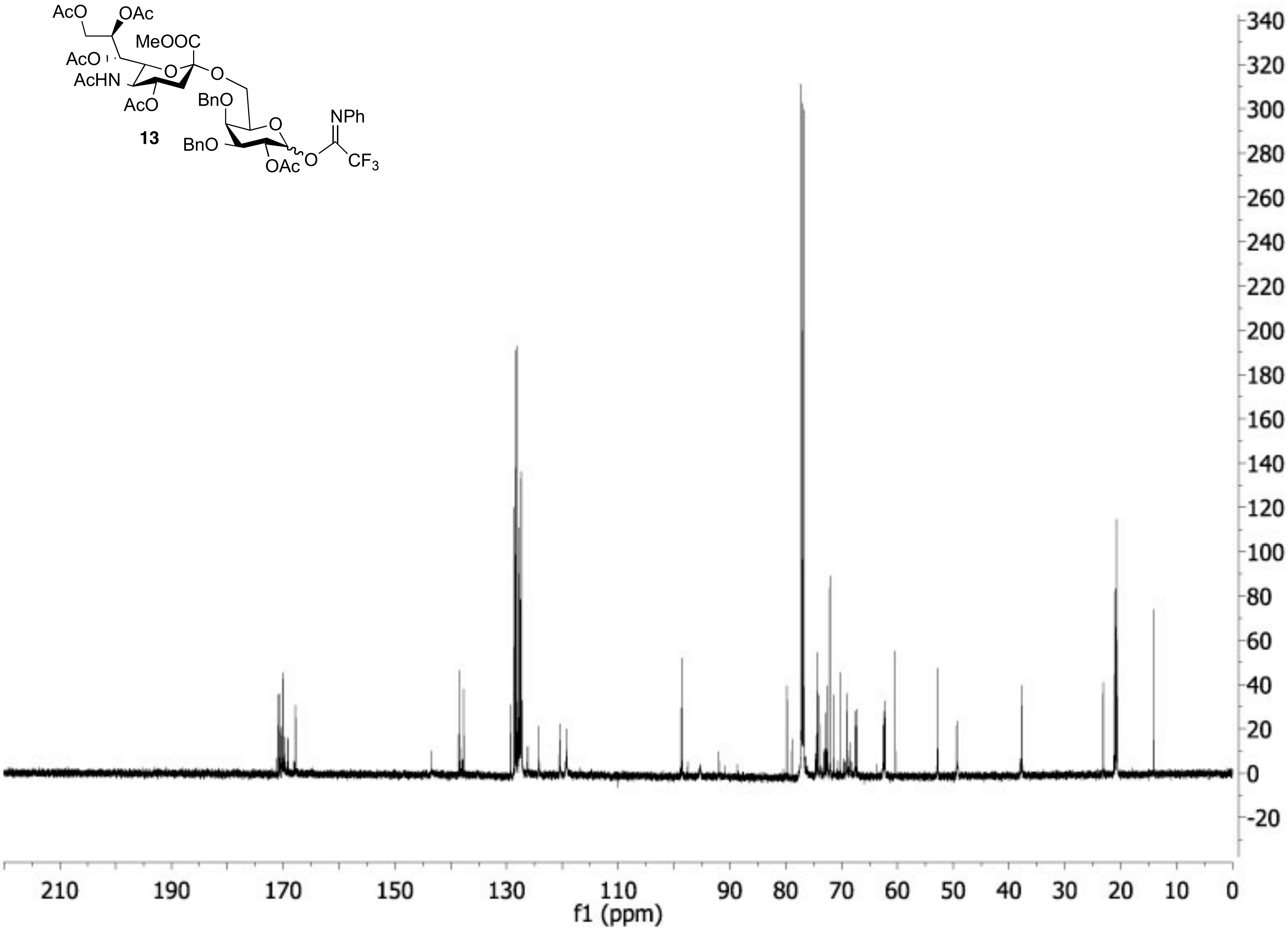
AcO
OAc
MeOOC
AcO
AcHN
AcO
BnO
BnO
NPh
OAc
CF3
13
f1 (ppm)

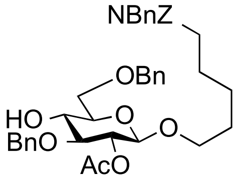
NBnZ
OBn
HO
O
O
BnO
AcO

14

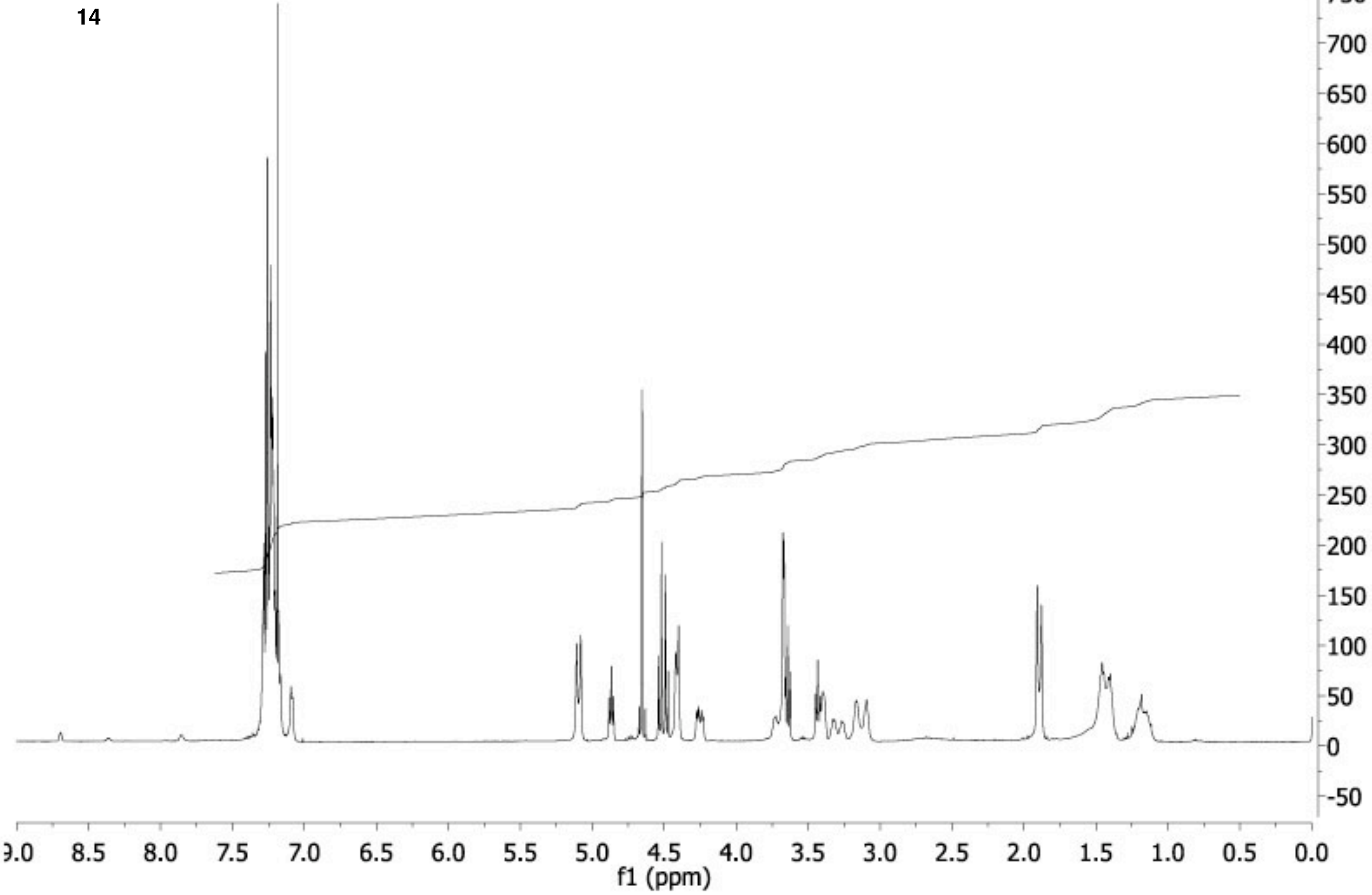
800
750
700
650
600
550
500
450
400
350
300
250
200
150
100
50
0
-50
8.5
8.0
7.5
7.0
6.5
6.0
5.5
5.0
4.5
4.0
3.5
3.0
2.5
2.0
1.5
1.0
0.5
0.0
f1 (ppm)

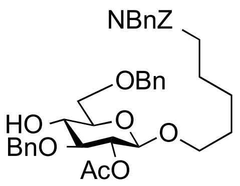
NBnZ
OBn
HO
O
O
BnO
AcO

14

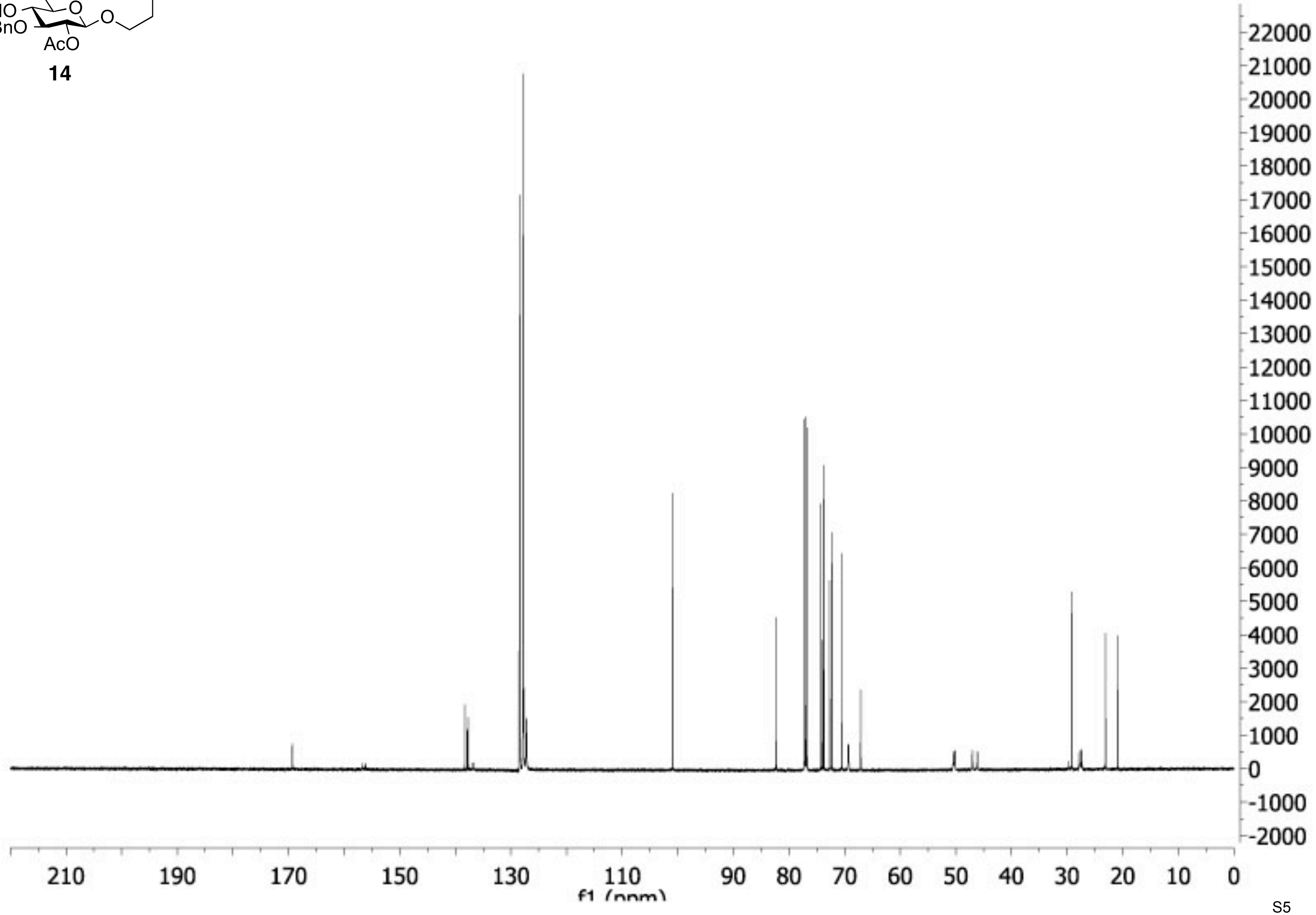
22000
21000
20000
19000
18000
17000
16000
15000
14000
13000
12000
11000
10000
9000
8000
7000
6000
5000
4000
3000
2000
1000
0
-1000
-2000
210 190 170 150 130 110 90 80 70 60 50 40 30 20 10 0
f1 (ppm)

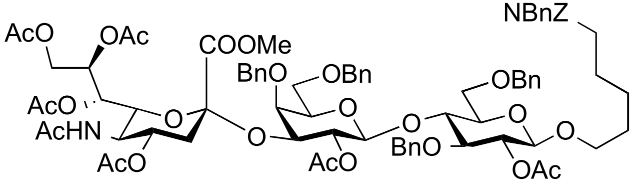
AcO
OAc
COOMe
BnO
OBn
NBnZ
OBn
AcO
AcHN
O
O
O
O
O
O
AcO
AcO
BnO
OAc

15

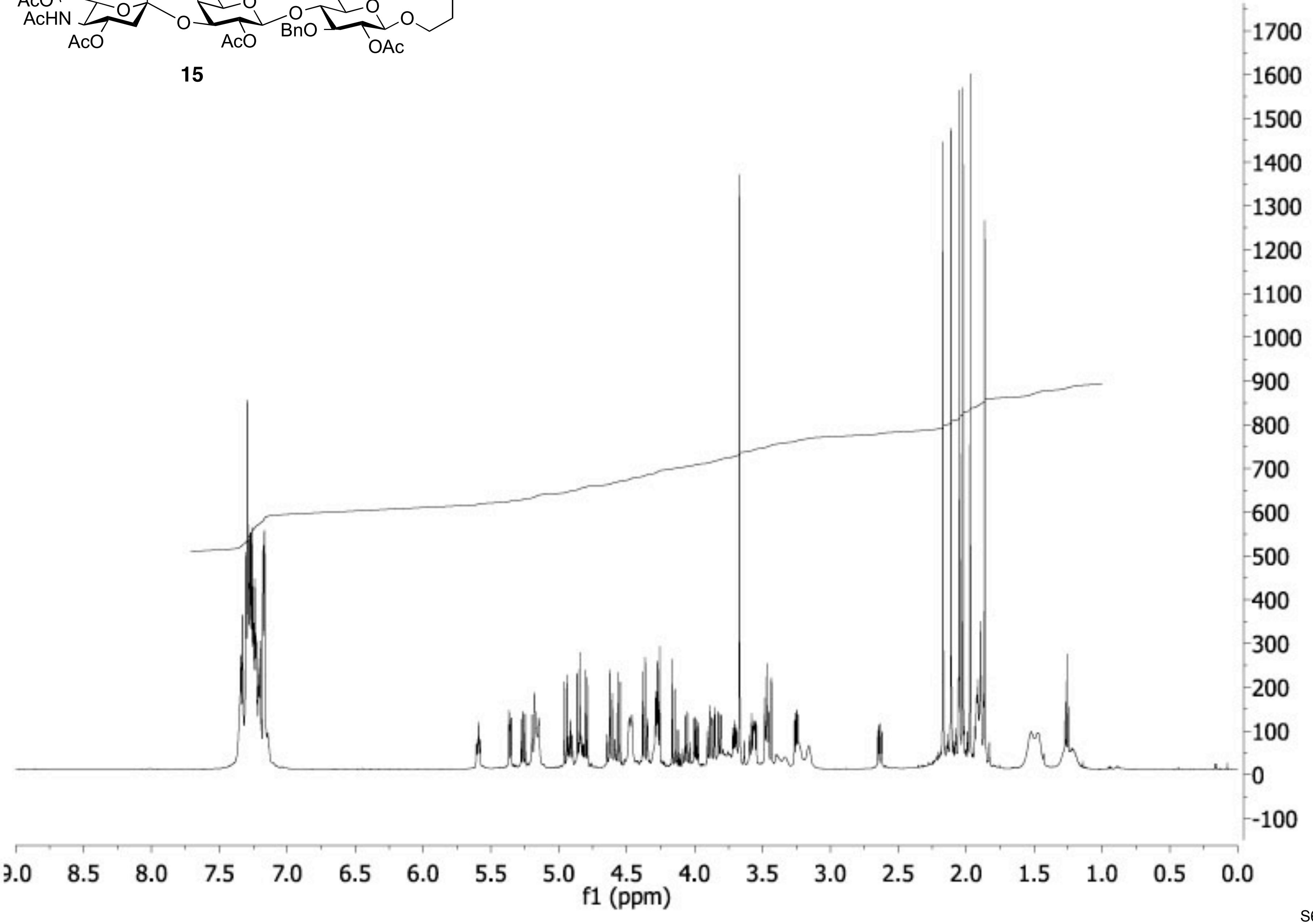
f1 (ppm)
9.0 8.5 8.0 7.5 7.0 6.5 6.0 5.5 5.0 4.5 4.0 3.5 3.0 2.5 2.0 1.5 1.0 0.5 0.0
1700 1600 1500 1400 1300 1200 1100 1000 900 800 700 600 500 400 300 200 100 0 -100

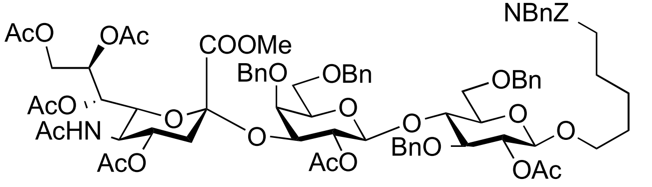
AcO
OAc
COOMe
NBnZ
BnO
OBn
OBn
AcO
AcHN
O
O
O
O
O
O
AcO
AcO
BnO
OAc

15

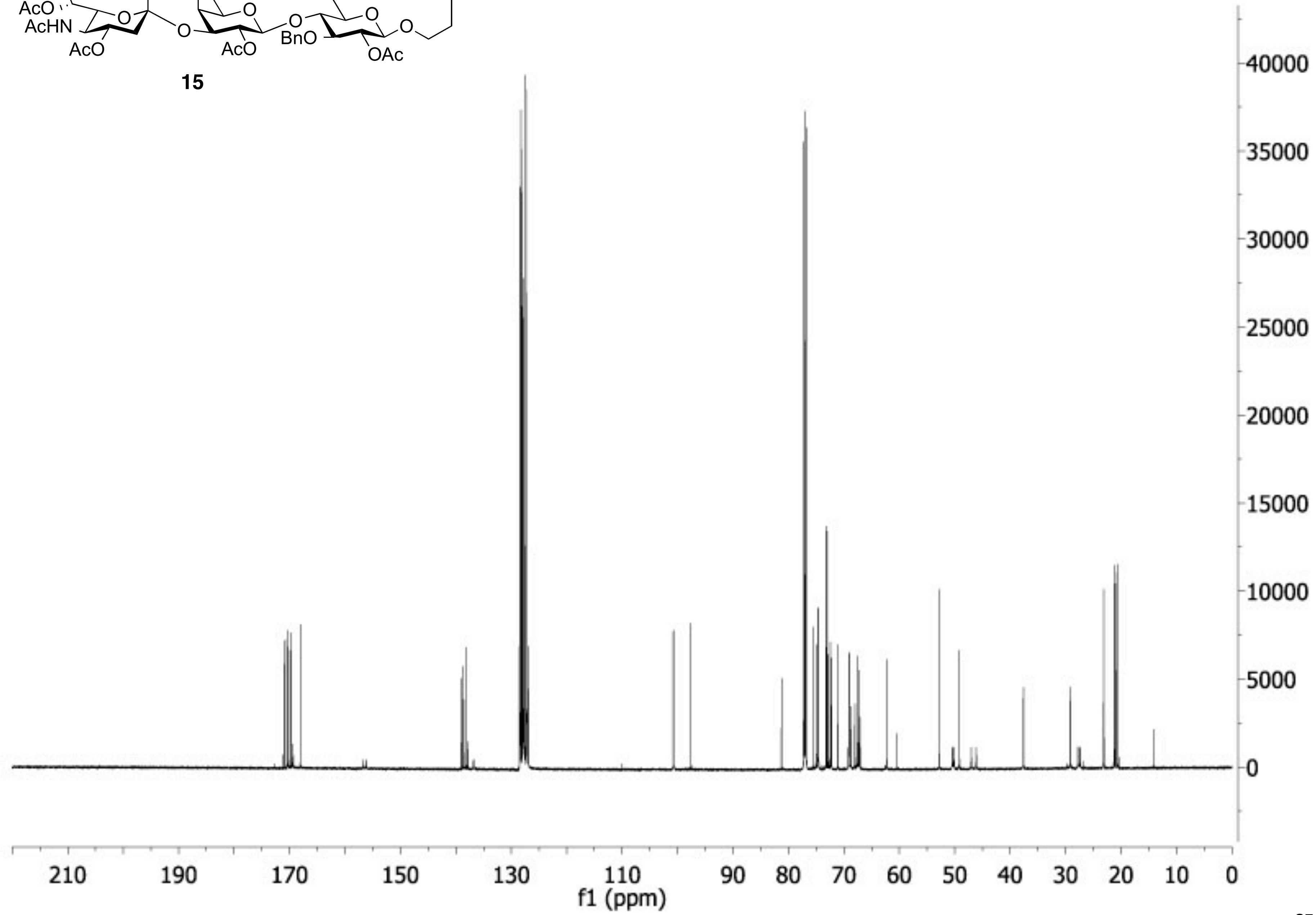
40000
35000
30000
25000
20000
15000
10000
5000
0
210
190
170
150
130
110
90
80
70
60
50
40
30
20
10
0
f1 (ppm)

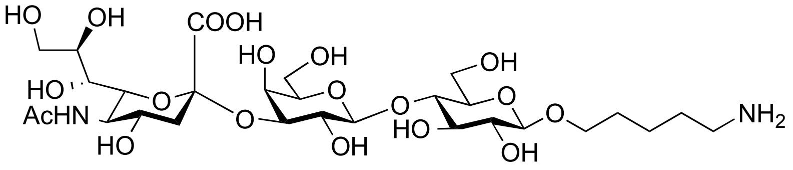
HO
OH
COOH
HO
OH
OH
HO
O
O
O
O
O
O
AcHN
HO
OH
HO
OH
NH2

16

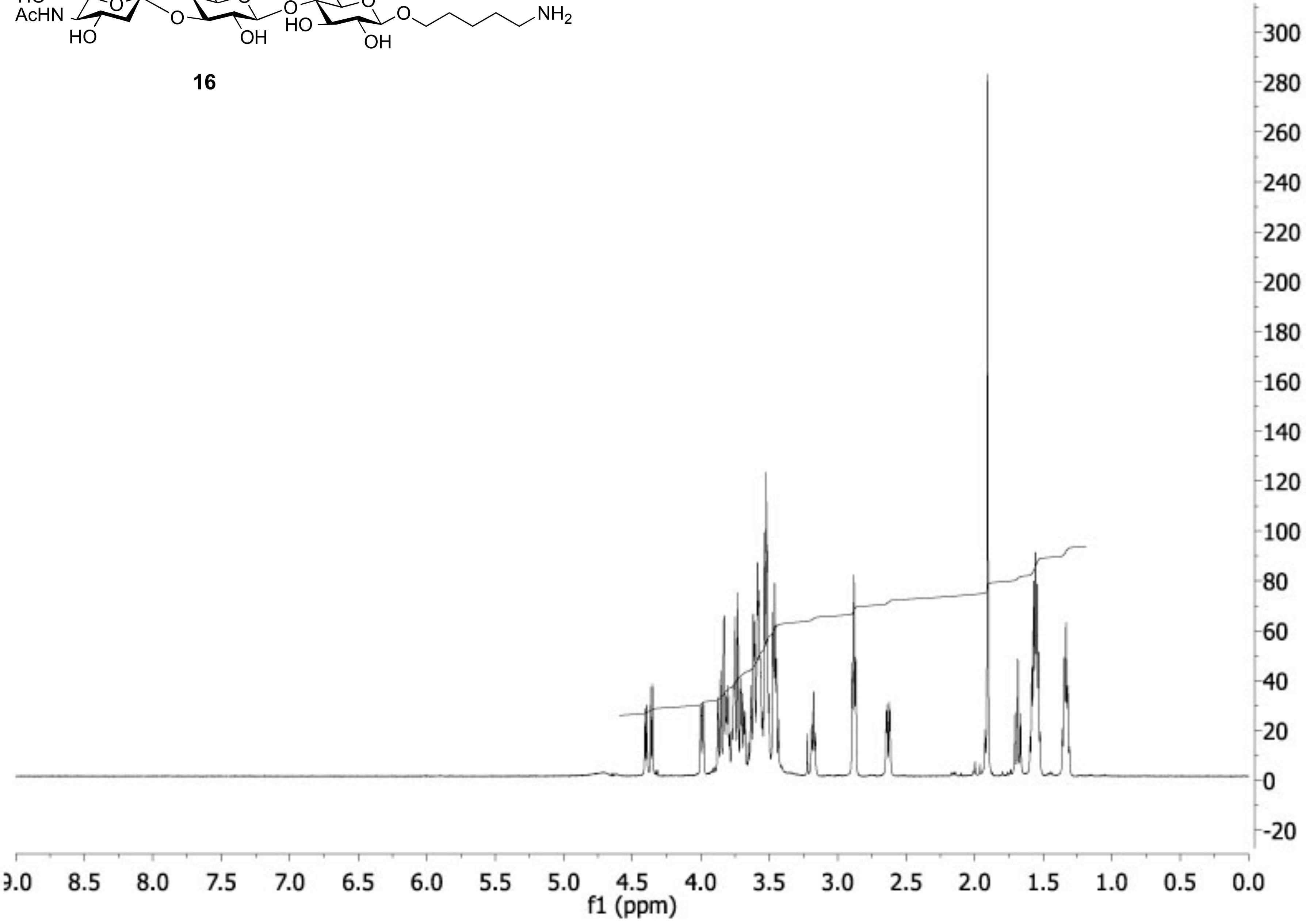
300
280
260
240
220
200
180
160
140
120
100
80
60
40
20
0
-20
9.0
8.5
8.0
7.5
7.0
6.5
6.0
5.5
5.0
4.5
4.0
3.5
3.0
2.5
2.0
1.5
1.0
0.5
0.0
f1 (ppm)

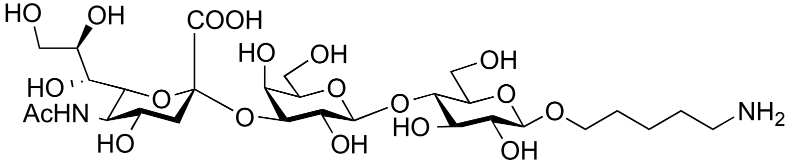
HO
OH
COOH
HO
OH
OH
HO
AcHN
O
O
O
O
O
O
HO
OH
HO
OH
NH2

16

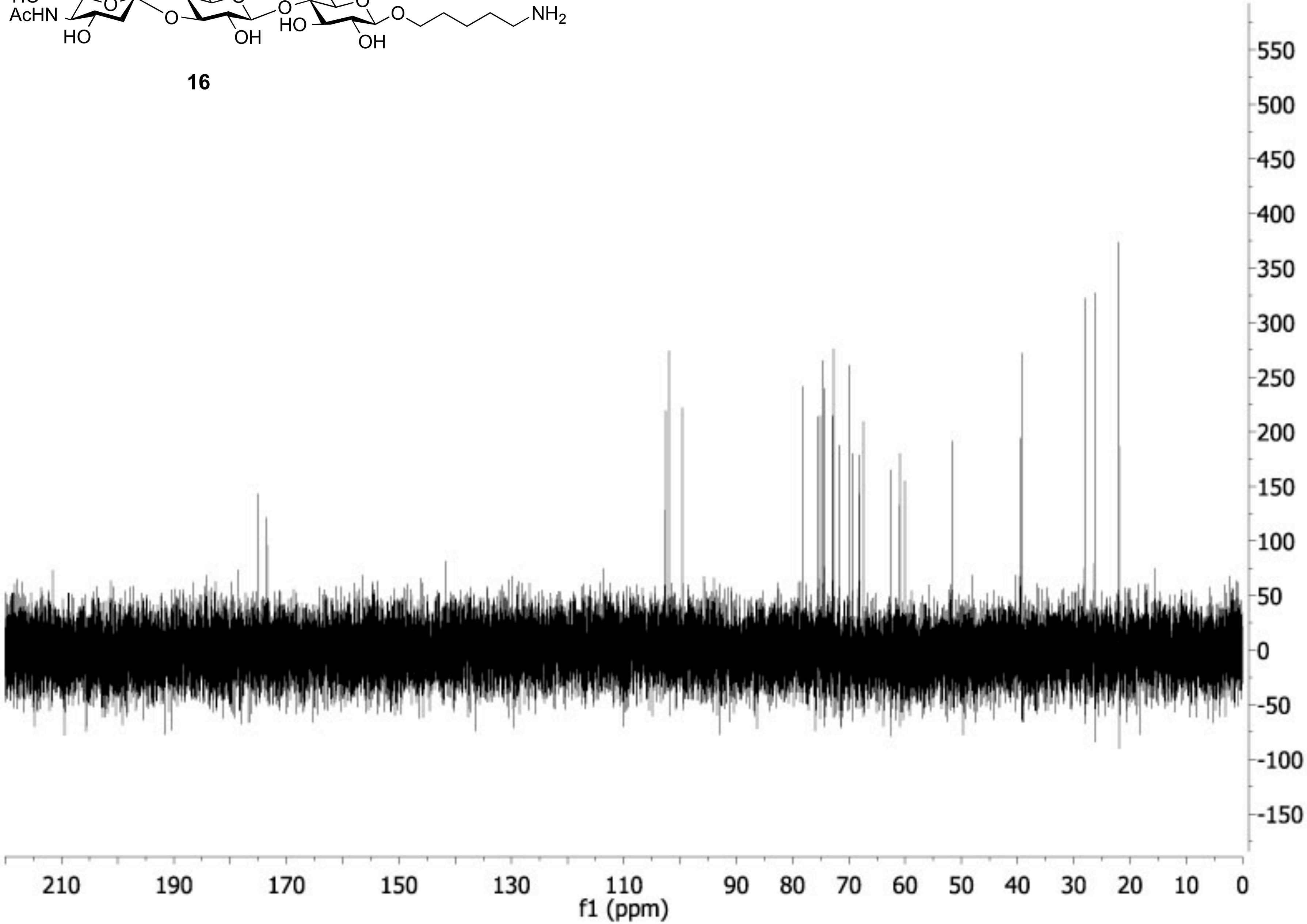
550
500
450
400
350
300
250
200
150
100
50
0
-50
-100
-150
210
190
170
150
130
110
90
80
70
60
50
40
30
20
10
0
f1 (ppm)

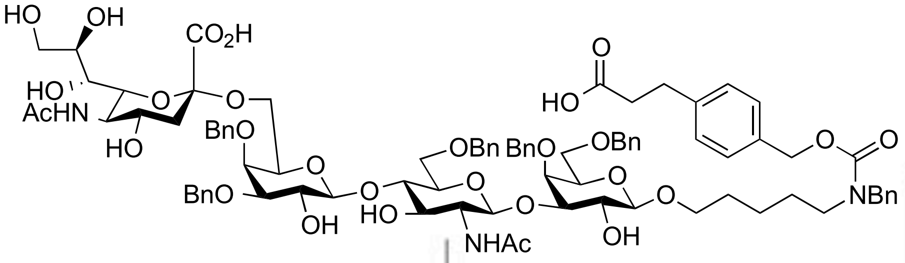

30

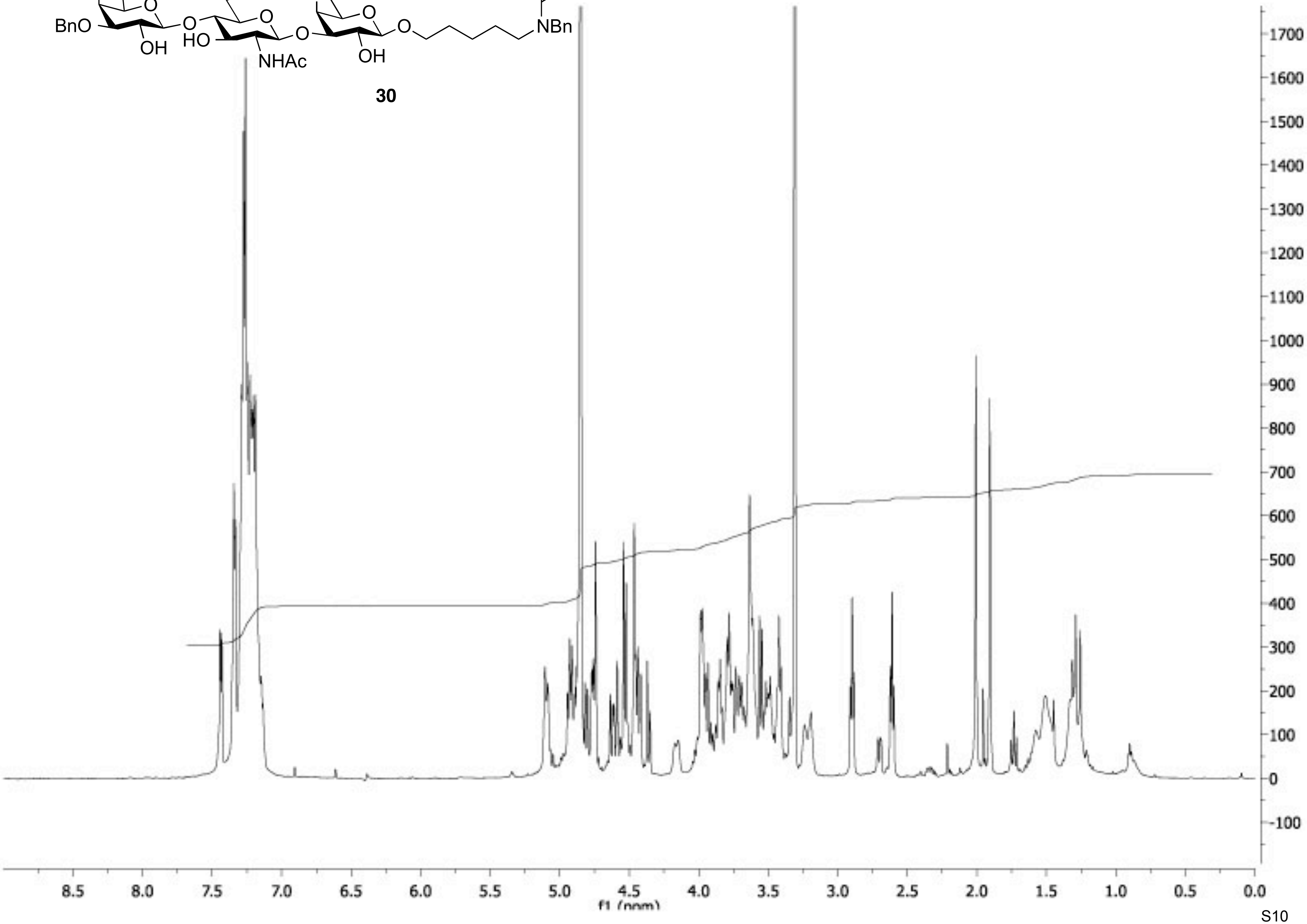

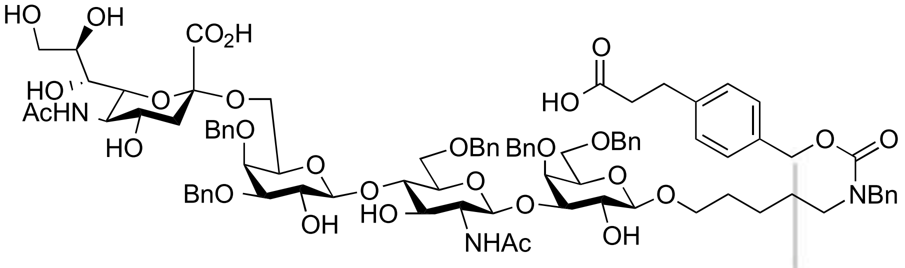
HO
OH
CO2H
HO
AcHN
O
O
HO
BnO
O
BnO
OH
O
OBn
BnO
OBn
O
HO
NHAc
O
OH
O
HO
O
O
O
NBn

30

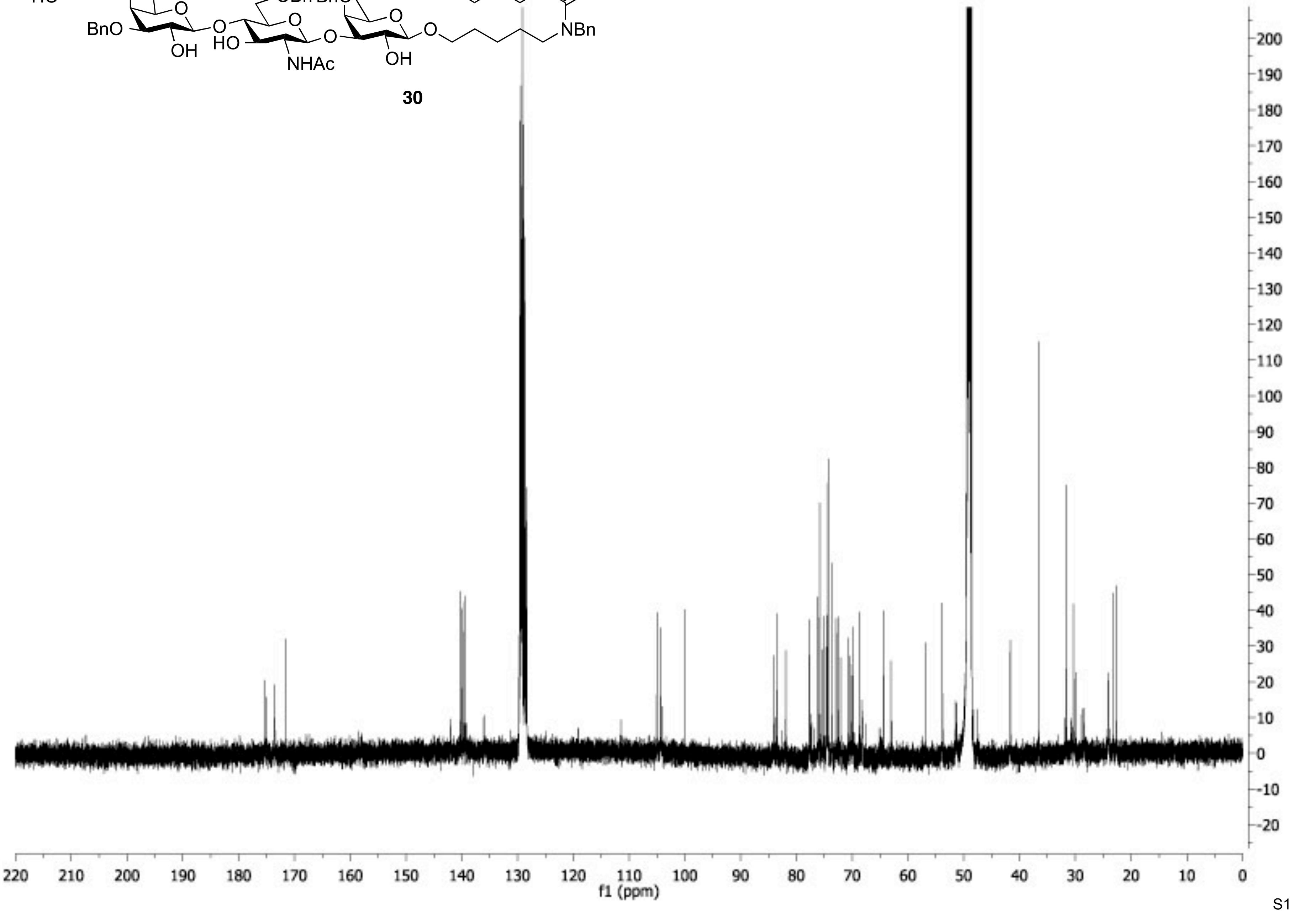
f1 (ppm)
220 210 200 190 180 170 160 150 140 130 120 110 100 90 80 70 60 50 40 30 20 10 0
200 190 180 170 160 150 140 130 120 110 100 90 80 70 60 50 40 30 20 10 0 -10 -20

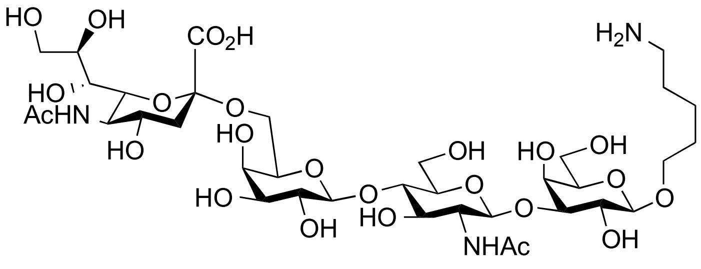
HO
OH
CO_2H
H_2N
HO
AcHN
O
HO
NHAc
OH

28

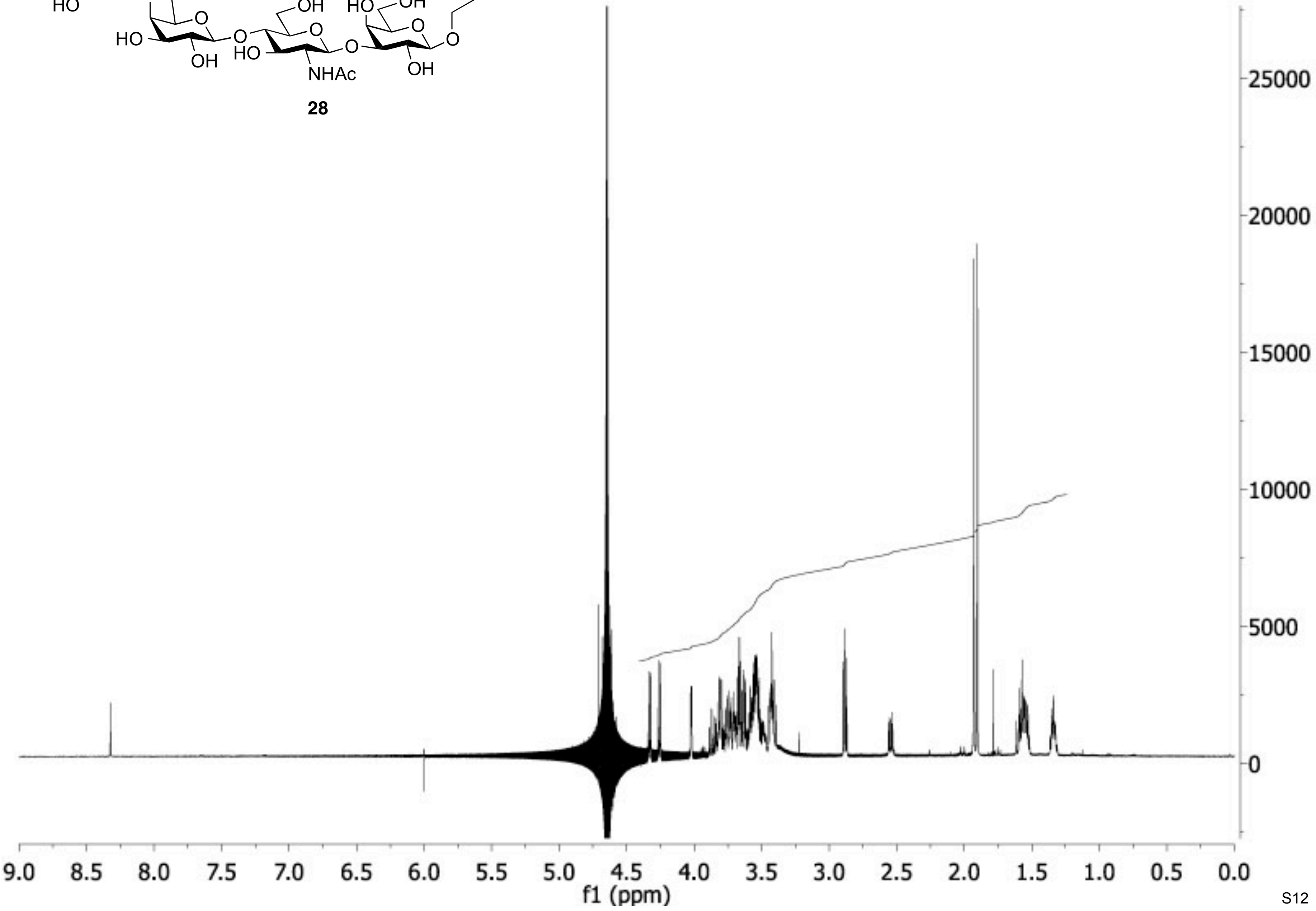
25000
20000
15000
10000
5000
0
9.0 8.5 8.0 7.5 7.0 6.5 6.0 5.5 5.0 4.5 4.0 3.5 3.0 2.5 2.0 1.5 1.0 0.5 0.0
f1 (ppm)

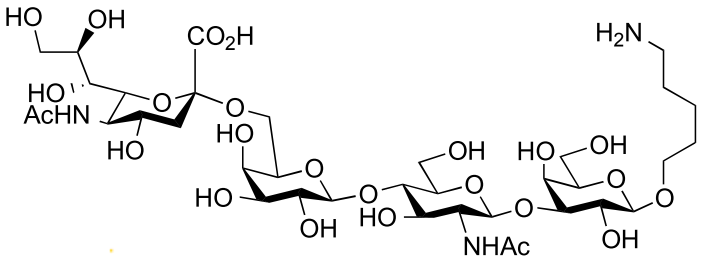

28

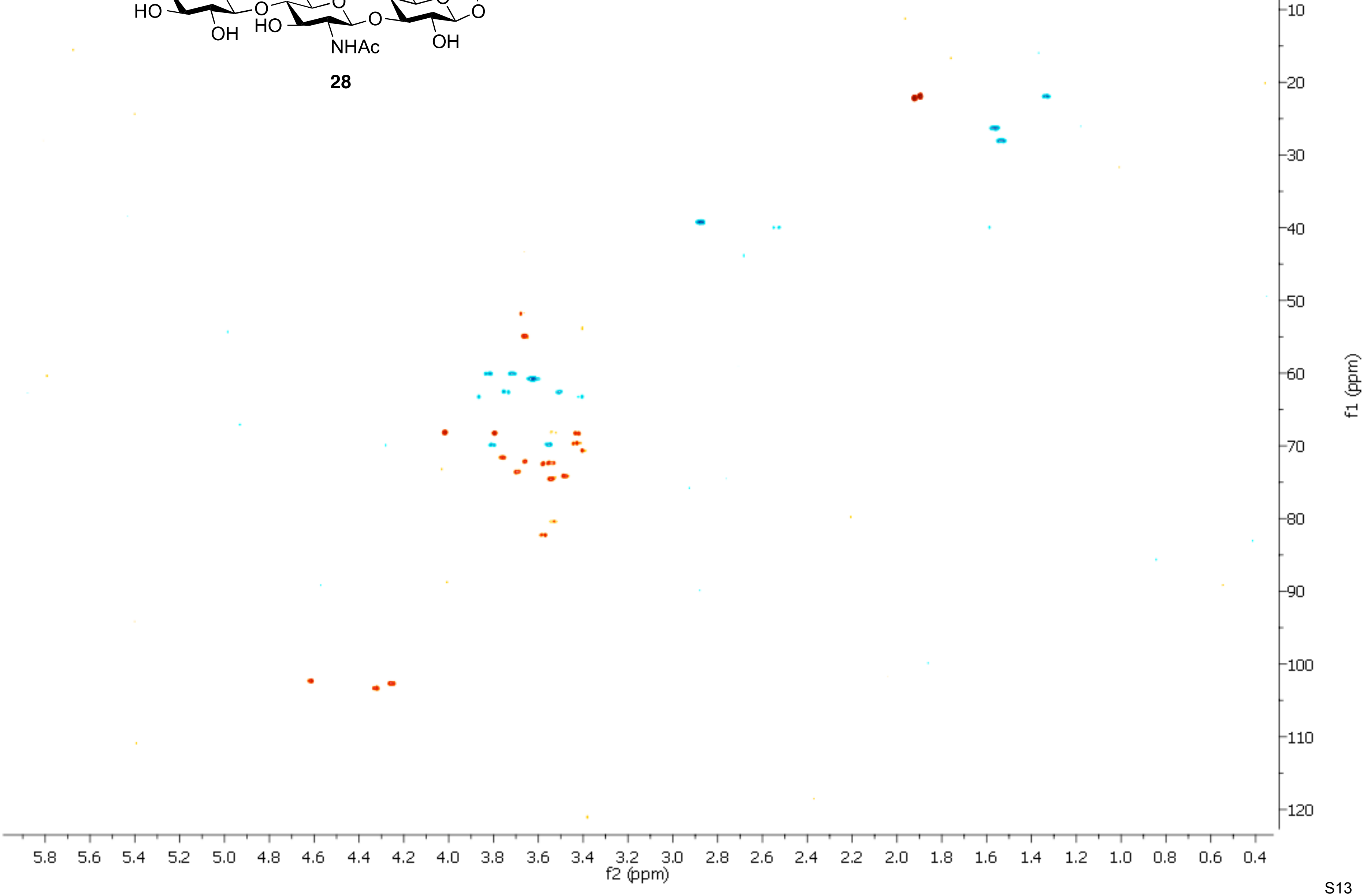
f1 (ppm)
10
20
30
40
50
60
70
80
90
100
110
120
5.8 5.6 5.4 5.2 5.0 4.8 4.6 4.4 4.2 4.0 3.8 3.6 3.4 3.2 3.0 2.8 2.6 2.4 2.2 2.0 1.8 1.6 1.4 1.2 1.0 0.8 0.6 0.4
f2 (ppm)

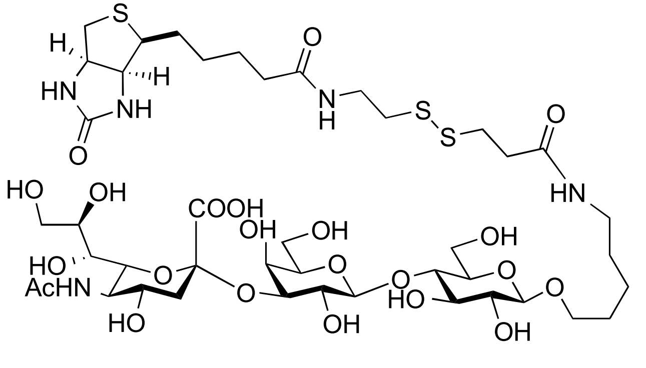

32

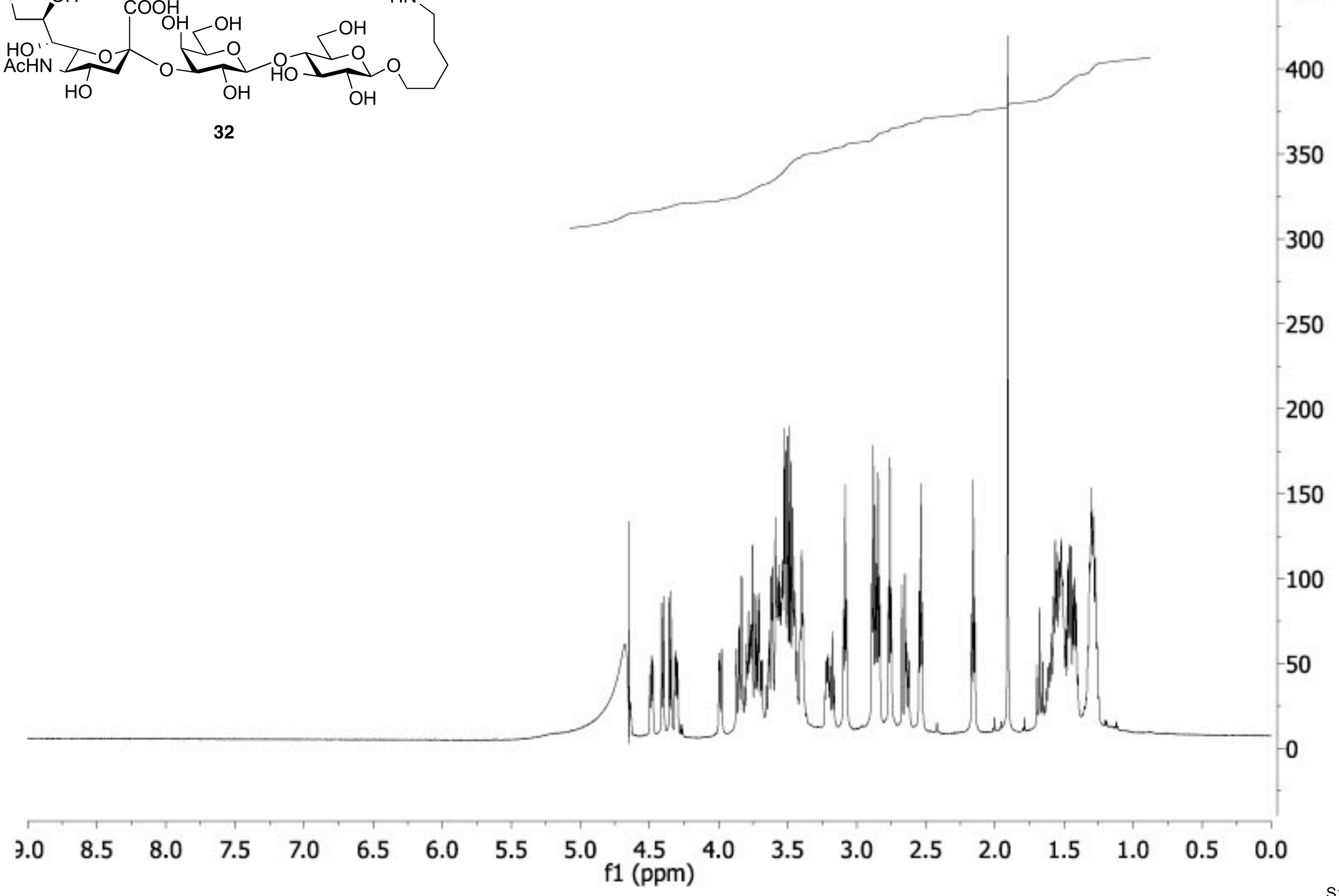

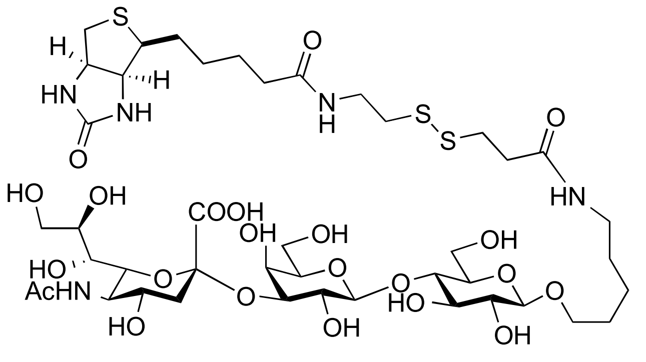

32

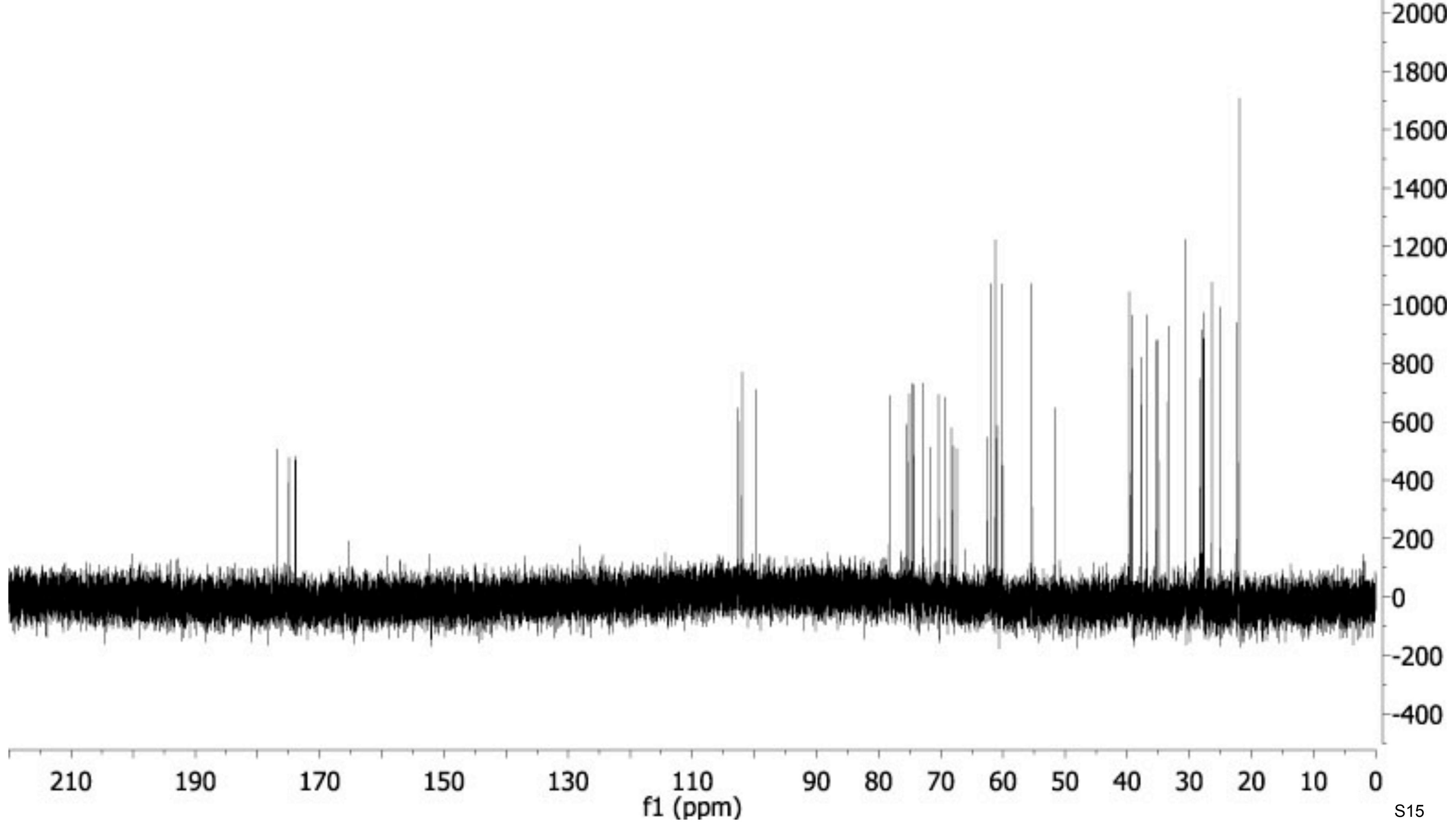

2600
2400
2200
2000
1800
1600
1400
1200
1000
800
600
400
200
0
-200
-400
210 190 170 150 130 110 90 80 70 60 50 40 30 20 10 0
f1 (ppm)
